# Hypothalamic volume is associated with dysregulated sleep in autistic and non-autistic young children

**DOI:** 10.1177/13623613251352249

**Published:** 2025-07-09

**Authors:** Burt Hatch, Derek Sayre Andrews, Brett Dufour, Shayan M Alavynejad, Joshua K Lee, Sally Rogers, Marjorie Solomon, Meghan Miller, Christine Wu Nordahl

**Affiliations:** 1 University of California, Davis, USA; 2 Victoria University of Wellington, New Zealand

**Keywords:** autism spectrum disorder, externalizing, hypothalamus, internalizing, MRI, sleep

## Abstract

Difficulty initiating or maintaining sleep is common among autistic individuals and co-occurs with internalizing and externalizing symptoms. This study tested associations between subcortical regions implicated in sleep processes and measures of dysregulated sleep initiation/maintenance in autistic and non-autistic 2- to 4-year-olds. The role of co-occurring externalizing and internalizing symptoms in these associations was also evaluated. Participants included 203 autistic (131 males, 72 females) and 92 non-autistic (49 males, 43 females) 2- to 4-year-olds who completed magnetic resonance imaging. A subscale of items from the Children’s Sleep Habits Questionnaire, previously shown to be reliable across both autistic and non-autistic children, was used to measure dysregulated sleep initiation/maintenance. Externalizing and internalizing symptoms were evaluated using the Child Behavior Checklist–Preschool. Associations between volumes for nine subcortical structures known to be implicated in sleep were separately modeled. Mediation analyses explored whether such associations could be accounted for by externalizing or internalizing symptoms. Smaller right hypothalamus volume was associated with dysregulated sleep initiation/maintenance in both autistic and non-autistic children. Externalizing (but not internalizing) problems partially mediated this association. Findings implicate the right hypothalamus in sleep initiation and maintenance issues for both autistic and non-autistic young children, supporting prior evidence of its central role in sleep regulation.

**Lay Abstract:**

Difficulty initiating or maintaining sleep is common among autistic individuals and often goes alongside difficulties regulating emotions and behavior during the day. Although there is a body of research suggesting that subcortical brain regions, including a brain region known as the hypothalamus, play important roles regulating sleep, few studies have examined whether this extends to young autistic children. Using data from a sample of 203 autistic (131 males, 72 females) and 92 non-autistic (49 males, 43 females) 2- to 4-year-olds, we examined whether size of subcortical brain regions implicated in sleep processes is associated with difficulties initiating and/or maintaining sleep. In addition, we examined whether daytime behaviors and emotions were also implicated in these associations. We found that smaller right hypothalamus volume was associated with dysregulated sleep initiation/maintenance in both autistic and non-autistic children. This relationship remained evident even after accounting for externalizing behaviors and emotions like anger that were also associated with both the hypothalamus and dysregulated sleep initiation/maintenance. The strength of association between right hypothalamus volumes and dysregulated sleep initiation/maintenance was similar for autistic and non-autistic children. These findings suggest that for both young autistic and non-autistic children, the hypothalamus plays unique roles in regulating both sleep and externalizing behaviors. For managing sleep initiation and maintenance difficulties in clinical practice, the findings underscore the importance of considering environmental (e.g. not having a regular bedtime routine) and neurobiological factors, for both autistic and non-autistic young children.

## Introduction

Autism spectrum disorder (ASD or autism), a neurodevelopmental condition characterized by difficulties with social communication and the presence of restricted and repetitive behaviors and interests (American Psychiatric Association, 2013), has an estimated prevalence of one in 36 individuals in the United States (Maenner et al., 2023). Although sleep difficulties, particularly difficulties initiating and maintaining overnight sleep, are common for non-autistic children (Steinsbekk et al., 2013; Williamson et al., 2019), autistic children tend to experience these difficulties more severely and/or more often (Carnett et al., 2021; Díaz-Román et al., 2018; Krakowiak et al., 2008; MacDuffie et al., 2020; Manelis-Baram et al., 2022; Richdale & Schreck, 2009). Indeed, evidence suggests autism is linked to difficulties initiating and maintaining sleep from at least toddlerhood and the preschool years (Humphreys et al., 2014; MacDuffie et al., 2020), when autism can first be reliably diagnosed (Barbaro & Dissanayake, 2017; Ozonoff et al., 2018). This is notable given that reduced sleep quantity and quality can negatively impact cognitive and academic abilities as well as exacerbate problem behaviors (Abel et al., 2018; Bernier et al., 2013; Calhoun et al., 2020; Cohen et al., 2014; Han et al., 2022; McLay et al., 2022; Sadeh et al., 2003; Vriend et al., 2013). The elevated impact of sleep disturbances among autistic children underscores a need to better understand underlying mechanisms, thus informing improvement of approaches to prevention and intervention (Beauchaine et al., 2008). Moreover, given that sleep disturbances affect not only autistic children, it is also important to investigate whether mechanisms underlying sleep problems are unique to autism or shared with non-autistic individuals, in order to understand how prevention or interventions targeting sleep might need to be tailored for autism.

Converging evidence suggests that sleep problems in autism, including difficulties initiating and maintaining sleep overnight, could be linked to alterations in neurobiological mechanisms that play distinct roles in sleep regulation, including circadian rhythm, sleep–wake homeostasis, and physiological arousal mechanisms (Ballester et al., 2020; Carmassi et al., 2019; Richdale & Schreck, 2009). For example, autism has been linked to variants in circadian-relevant genes (Yang et al., 2016), and less melatonin, a hormone that regulates sleep–wake timing (Melke et al., 2008). In terms of brain structures, the neurobiological mechanisms regulating sleep rely on interconnected neural circuits that predominantly lie within subcortical regions, particularly the hypothalamus, thalamus, amygdala, hippocampus, globus pallidus, basal forebrain, and brainstem (Adamantidis et al., 2019; Coenen, 2024; Mitra et al., 2016; Mizrahi-Kliger et al., 2018; Saper et al., 2010; Scammell et al., 2017; Yamashita & Yamanaka, 2017). However, the extent that these regions are associated with difficulties initiating and maintaining sleep in autistic children is not well understood.

Brain imaging studies of non-autistic children have demonstrated that reduced volume in several subcortical regions is associated with greater difficulty initiating or maintaining sleep (Hernandez et al., 2023), reduced sleep duration (Cheng et al., 2021; Riggins & Spencer, 2020; Taki et al., 2012), or increased sleep problems as measured by a composite of difficulties initiating and maintaining sleep, sleep breathing troubles, and excessive somnolence (Shen et al., 2020). For example, data from the Adolescent Brain Cognitive Development (ABCD) study have demonstrated in a large community sample of 9- to 11-year-olds that although reduced volume of multiple brain regions predicts parent-reported shorter sleep duration and difficulties initiating and maintaining sleep, these associations are strongest for the hippocampus, amygdala, caudate nucleus, nucleus accumbens, and thalamus (Cheng et al., 2021; Hernandez et al., 2023). Few studies have investigated associations between brain morphology and sleep in autism. One magnetic resonance imaging (MRI) study found that the Children’s Sleep Habits Questionnaire (CSHQ) total scores, which reflects a range of different sleep problems (e.g. sleep onset, parasomnia, sleep anxiety, sleep-disordered breathing), tend to be higher among autistic 24- to 42-month-old children with elevated extra-axial cerebrospinal fluid (Shen et al., 2018). Another study investigated associations between subcortical morphology and sleep problems across both autistic and non-autistic children (MacDuffie et al., 2020). This study followed infants at high or low familial likelihood for autism and measured parent-reported sleep onset difficulties when initiating sleep or after waking (e.g. hard time settling down to sleep, not often able to go back to sleep immediately after waking) when children were 6 and 12 months of age, along with MRI at 6, 12, and 24 months of age. Increased sleep initiation problems were associated with increased hippocampal volume at 6, 12, and 24 months of age for autistic children. However, for non-autistic children, sleep initiation was not associated with any of the subcortical brain regions examined. These findings are inconsistent with the aforementioned pattern of increased sleep difficulties associated with reduced subcortical volumes demonstrated in studies of older non-autistic children (Cheng et al., 2021; Hernandez et al., 2023; Riggins & Spencer, 2020; Shen et al., 2020; Taki et al., 2012). These inconsistent findings may be accounted for by methodological differences across studies, including age differences between autistic and non-autistic samples, but could also reflect that at least some neurobiological mechanisms contributing to dysregulated sleep initiation or maintenance are unique to autism (Kamara & Beauchaine, 2020). Disentangling the extent that neurobiological mechanisms underlying sleep difficulties might be unique to autism or not has implications for improving targeted approaches to intervention, however, empirical investigations with such aims have several considerations to address.

One challenge is that measures of sleep problems, including difficulties initiating or maintaining overnight sleep, may not be equally reliable across different groups (Hatch et al., 2021; Mancini et al., 2019), in which case groupwise comparisons on the scale would be invalid (Meredith, 1993). Previous studies have demonstrated that parent-report scales typically used to assess sleep differ significantly in reliability in autistic children (as well as those with other neurodevelopmental conditions) compared with typically developing children (Hatch et al., 2021; Mancini et al., 2019). In a study of autistic and non-autistic 2- to 4-year-olds, we investigated the factor structure of the CSHQ, a measure widely used in both research and clinical practice, and demonstrated differences in the way items related to sleep constructs for autistic and non-autistic children (Hatch et al., 2021). Accordingly, to validly compare autistic and non-autistic children on the magnitude of associations between subcortical volumes and dysregulated sleep initiation/maintenance, it is critical to use a measure of dysregulated sleep initiation/maintenance that is equally reliable across samples.

In addition, although multiple studies have demonstrated that difficulties initiating or maintaining sleep correlate with externalizing (e.g. inattention, impulsivity, overactivity) and internalizing (e.g. anxiety or depression) symptoms (Carmassi et al., 2019; Cohen et al., 2014; Coto et al., 2018; Gregory & Sadeh, 2016; Mazurek & Petroski, 2015; Quach et al., 2018), it remains unclear what this means in the context of neurobiological underpinnings. In part, externalizing and internalizing behaviors may either directly contribute to poor sleep or arise from poor sleep. In addition, data from non-autistic samples suggest that externalizing or internalizing symptoms might co-occur with sleep difficulties because of common neurobiological mechanisms (Cheng et al., 2021; Shen et al., 2020). For example, reduced volume in subcortical regions (insula, caudate, putamen, hippocampus, amygdala) is associated with both attention-deficit/hyperactivity disorder (ADHD) symptom severity and a composite of sleep problems (disorders of initiating and maintaining sleep, sleep breathing disorders, and disorders of excessive somnolence) have been found to be partially mediated by the severity of ADHD symptoms (Shen et al., 2020). Understanding whether associations between regional brain volumes and dysregulated sleep initiation/maintenance are mediated by externalizing or internalizing symptoms could further clarify the extent to which these sleep problems arise from similar or distinct neurobiological mechanisms in both autistic and non-autistic individuals.

The primary aim of this study was to explore possible associations between volumes of subcortical brain structures involved in sleep processes and dysregulated sleep initiation/maintenance among autistic and non-autistic 2- to 4-year-olds. Critically, this study utilizes a measure of dysregulated sleep initiation/maintenance that is equally reliable for both autistic and non-autistic children (Hatch et al., 2021). Based on limited previous findings, we hypothesized that associations between subcortical volumes and dysregulated sleep initiation/maintenance would differ by group (autistic or non-autistic). Specifically, we expected that reduced subcortical volumes would be associated with increased dysregulation of sleep initiation/maintenance in non-autistic children but that the magnitude or direction of these associations would differ for autistic individuals. In addition, we aimed to investigate whether associations between subcortical volumes and dysregulated sleep initiation/maintenance could be accounted for (mediated) by co-occurring externalizing and internalizing symptoms.

## Methods

### Participants

This study included 203 autistic (131 males, 72 females) and 92 non-autistic (49 males, 43 females) 2- to 4-year-old children (Table 1). Participants were enrolled in the UC Davis MIND Institute Autism Phenome Project (APP), which includes the Girls with Autism: Imaging of Neurodevelopment (GAIN) and Brain Research in Autism Investigating Neurophenotypes (BRAIN) studies (Nordahl et al., 2022). All children from these cohorts with structural MRI scans of sufficient quality and a completed CSHQ at the time of study entry were included (Owens et al., 2000). ASD diagnosis was confirmed by research-reliable clinical psychologists using the Autism Diagnostic Observation Schedule–Generic (ADOS-G; Lord et al., 2000) or ADOS-2 (Lord et al., 2012), the Autism Diagnostic Interview–Revised (ADI-R; Rutter et al., 2003), and application of *Diagnostic and Statistical Manual of Mental Disorders* (4th ed., text rev.; *DSM-IV-TR*; American Psychiatric Association, 2000) or *Diagnostic and Statistical Manual of Mental Disorders* (5th ed.; *DSM-5*; American Psychiatric Association, 2013) criteria. Informed consent was obtained from the parent or guardian of each participant. All aspects of the study were approved by the University of California, Davis Institutional Review Board. See Supplementary methods for additional details.

**Table 1.** Participant characteristics.

	ASD (*n* = 203)	Non-ASD (*n* = 92)	Statistic
Sex, female, *n* (%)	72 (35%)	43 (46%)	χ^2^= 3.38, *p* = 0.06
Age (months)	38.06 (5.47)	37.35 (6.10)	*t* = -0.94, *p* = 0.34
Ethnicity, Hispanic, *n* (%)	49 (24%)	26 (28%)	χ^2^= 0.57, *p* = 0.75
Race, *n* (%)			χ^2^= 7.94, *p* = 0.08
African American/Black	9 (4%)	2 (2%)	
Asian	21 (10%)	2 (2%)	
Mixed/Other	36 (18%)	17 (18%)	
Refused/Not reported	10 (5%)	3 (3%)	
White/Caucasian	127 (63%)	68 (74%)	
Income, *n* (%)			χ^2^= 2.82, *p* = 0.08
Less than $100,000	124 (61%)	48 (52%)	
$100, 000 to $149,999	25 (12%)	25 (27%)	
$150,000 and above	34 (17%)	16 (52%)	
DQ	65.19 (22.12)	106.15 (12.84)	*t* = 19.93, *p* < 0.001
ADOS-CSS	7.4 (1.78)	1.3 (0.57)	*t* = 38.32, *p* < 0.001
Dysregulated Sleep	11.65 (3.03)	10.03 (1.99)	*t* = 5.42, *p* < 0.001
CSHQ Total	45.74 (8.22)	42.37 (6.60)	*t* = 3.73, *p* < 0.001
CBCL Externalizing	59.63 (10.44)	46.89 (9.25)	*t* = 10.23, *p* < 0.001
CBCL Internalizing	62.41 (8.94)	42.97 (9.18)	*t* = 16.28, *p* < 0.001

Dysregulated Sleep is the dysregulated sleep initiation/maintenance scale derived from the CSHQ (Hatch et al., 2021). CSHQ Total is the original composite of items as per Owens et al. (2000). While data presented by Owens et al. (2000) suggest a cutoff of 41 on the CSHQ Total indicates children with sleep problems, this is based on data from 4- to 10-year-olds and may not apply to this younger cohort. Ethnicity not collected for *n* = 3 from non-ASD and *n* = 7 for ASD. Income not collected for *n* = 3 non-ASD and *n* = 20 for ASD. DQ not collected for *n* = 1 non-ASD. ADOS not collected from *n* = 57 non-ASD. CSHQ Total not collected from *n* = 1 non-ASD and *n* = 2 ASD. CBCL Externalizing not collected from *n* = 4 non-ASD and *n* = 15 ASD. CBCL Internalizing not collected from *n* = 7 non-ASD and *n* = 18 ASD. Non-ASD and ASD did not differ on proportion of missing data for Ethnicity, Race, Income, DQ, CSHQ Total, CBCL Externalizing, and CBCL Internalizing. ASD = autism spectrum disorder; DQ = Developmental Quotient; ADOS-CSS = Autism Diagnostic Observation Scale–Calibrated Severity Score; CSHQ = Children’s Sleep Habits Questionnaire; CBCL = Child Behavior Checklist.

### Dysregulated sleep initiation/maintenance scale

Dysregulated sleep initiation/maintenance was measured using a scale composed of seven items from the parent-reported CSHQ (Owens et al., 2000). Although CSHQ items have demonstrated validity (e.g. correlate with other sleep measures, differentiate children with/without sleep problems), the original item composites do not reliably measure the same sleep-related constructs in autistic children (Hatch et al., 2021). The seven-item composite of dysregulated sleep initiation/maintenance (*Asleep within 20* *min after bed* (reverse scored), *Sleeps same each day* (reverse scored), *Sleeps too little*, *Goes to bed same time at night*, *Awakes more than once*, *Restless and moves during sleep*, and *Child seems tired*) has been demonstrated to reflect this construct for both autistic and non-autistic children with comparable reliability (Hatch et al., 2021). Accordingly, this composite is suited to research involving comparisons between autistic and non-autistic children. However, we also conducted Supplementary analyses using the original version of the CSHQ total scale. On average, compared with non-autistic participants, autistic participants were significantly higher on dysregulated sleep initiation/maintenance (*t* = 5.42, *p* < 0.001) and the original CSHQ total scale (*t* = 3.73, *p* < 0.001).

### Externalizing and internalizing symptoms

The parent-reported Child Behavior Checklist (CBCL)–1.5-5 (Preschool) form (Achenbach & Rescorla, 2000) was used to measure externalizing and internalizing symptoms. The CBCL consists of items (each with three response options) covering a variety of emotional and behavioral problems. Item composites provide scores for empirically derived syndromes which in turn comprise scores for Internalizing and Externalizing symptoms. Composite scores can be transformed to age and sex standardized *T* scores (*M* = 50, *SD* = 10). For sample descriptives, we present *T* scores; for main analyses testing mediation effects, we used raw composite scores given models also controlled for effects of age and sex.

### MRI acquisition and region of interest approach

**Figure 1.**
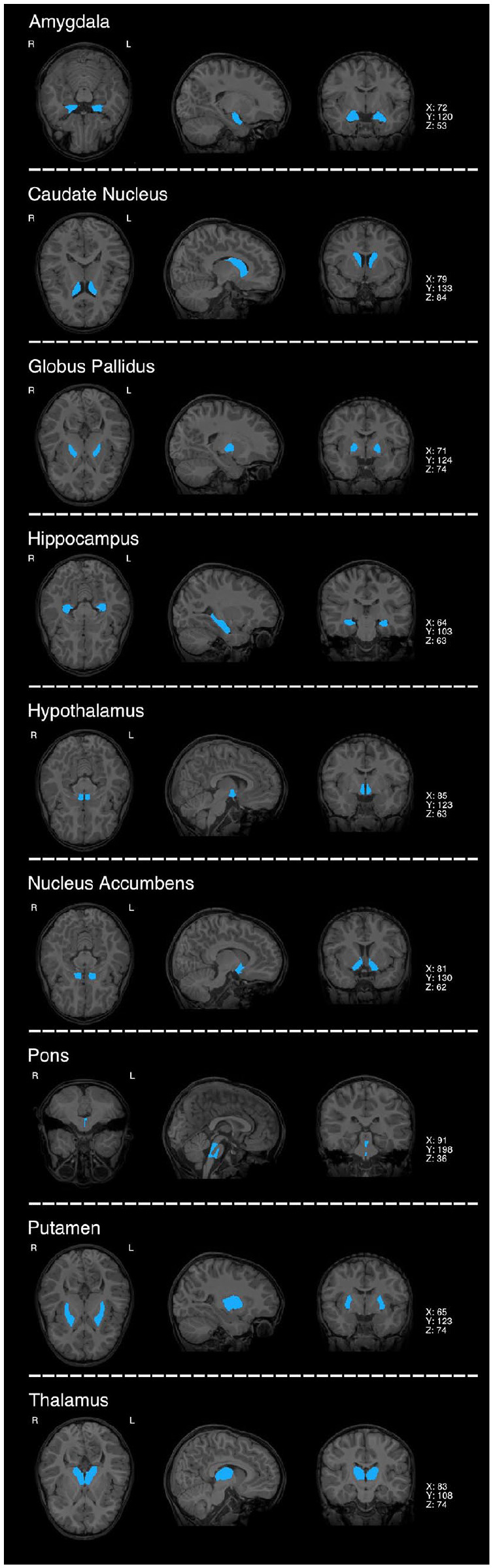
Regions of interest implicated in sleep regulation. A set of nine bilateral regions of interest (ROI) previously implicated in sleep regulation are highlighted for a single random participant in standard MNI space. Regions were defined using a multi-atlas approach in using the fully automated MRICloud T1-Segmentation pipeline v7A. Images are displayed in radiological right/left convention. MNI x y z coordinates at which each ROI image set was captured are provided.

MRI scans were acquired during natural nocturnal sleep at UC Davis Research Imaging Center from 2006 to 2019 on a 3T Siemens Trio whole-body MRI system using an eight-channel head coil. High-resolution, T1-weighted structural scans were acquired using a 3-dimensional magnetization-prepared rapid acquisition gradient-echo (MPRAGE) sequence. MPRAGE images were then segmented into 289 anatomically defined regions using an age-specific multi-atlas approach in the fully automated MRICloud (https://mricloud.org) T1-Segmentation pipeline v7A (Mori et al., 2016; Wang et al., 2013). Whole-brain segmentation output for each participant was downloaded and visually inspected for segmentation quality. Volumes were extracted and exported for further statistical analysis from nine a priori regions of interest (ROIs; Mitra et al., 2016; Mizrahi-Kliger et al., 2018; Saper et al., 2010; Scammell et al., 2017): the left and right hypothalamus, thalamus, amygdala, caudate nucleus, putamen, hippocampus, nucleus accumbens, globus pallidus, and pons (illustrated in Figure 1). See Supplementary methods for additional details.

### Statistical modeling

Effects of nine volumetric ROIs (hypothalamus, hippocampus, thalamus, amygdala, nucleus accumbens, pons, caudate nucleus, globus pallidus, and putamen) on dysregulated sleep initiation/maintenance were tested in turn by linear regression models using the “stats” package in *R* version 3.6 (R Core Team, 2019). Each model included sex, age in months, and total cerebral volume as covariates, and an interaction term between diagnosis and both left and right regional volumes:

$$\begin{array}{l} Y\;=\;\beta 1\;Left\;ROI\;*\;Diagnosis\;\\ \;\;\;\;\;\;\;\;\;\;+\;\beta 2\;Right\;ROI\;*\;Diagnosis\;\\ \;\;\;\;\;\;\;\;\;\;+\;\beta 3\;Sex\;+\;\beta 4\;Age\;+\;\beta 5\;TCV\;+\;E, \end{array}$$

where *E* is residual error. Inclusion of additional interaction effects between regional volumes, sex, and diagnosis were tested in a step-down fashion by likelihood ratio tests. *P* values were adjusted for multiple testing using a false discovery rate approach (FDR) across all ROIs tested (*n* = 18 tests; Benjamini & Hochberg, 1995),

### Mediation analyses

Mediation analyses were conducted to explore whether any associations identified between regional subcortical volumes and dysregulated sleep initiation/maintenance could be accounted for by externalizing or internalizing symptoms. Mediation analyses were conducted following the product-of-coefficients test using the “lavaan” package (Russeel, 2012) in R version 3.6 (R Core Team, 2019). First, separate regression analyses were conducted to examine whether regional brain volumes found to be significantly associated with dysregulated sleep initiation/maintenance were also significantly associated with externalizing or internalizing symptoms (path *a* effect). Second, if regional brain volume was significantly associated with either potential mediator, that is internalizing or externalizing symptoms, additional regression analyses were conducted to examine whether these mediators were associated with dysregulated sleep initiation/maintenance (path *b* effect). Finally, if both *a* and *b* effects were significant, mediation was tested using 2000 Monte Carlo simulations to obtain 95% confidence intervals to infer significance of the product-of-coefficients from *a* and *b* (Preacher & Selig, 2012). Analyses were first conducted averaging across autistic and non-autistic groups before exploring group moderated mediation effects. Because of missing data for externalizing (6.5%) and internalizing (9.5%) symptoms, analyses were conducted on 200 multiply imputed data sets, with regression estimates pooled following Rubin’s (1987) rules. Consistent with the primary analyses predicting sleep problems, each model included fixed effects for sex, total cerebral volume, and age in months. For further details of multiple imputation, see the Supplementary Methods. MRI and behavioral data from the APP are available through the National Database for Autism Research (NDAR). Complete data associated with this publication can be provided by the authors upon request. R Markdown template scripts to replicate the statistical analyses conducted within this study are provided within Supplementary materials.

### Community involvement

At the initiation of the APP, a series of focus groups were conducted with parents of autistic children to get their input on study design and overarching questions.

## Results

### Association of subcortical brain volumes with dysregulated sleep initiation/maintenance scores

**Figure 2.**
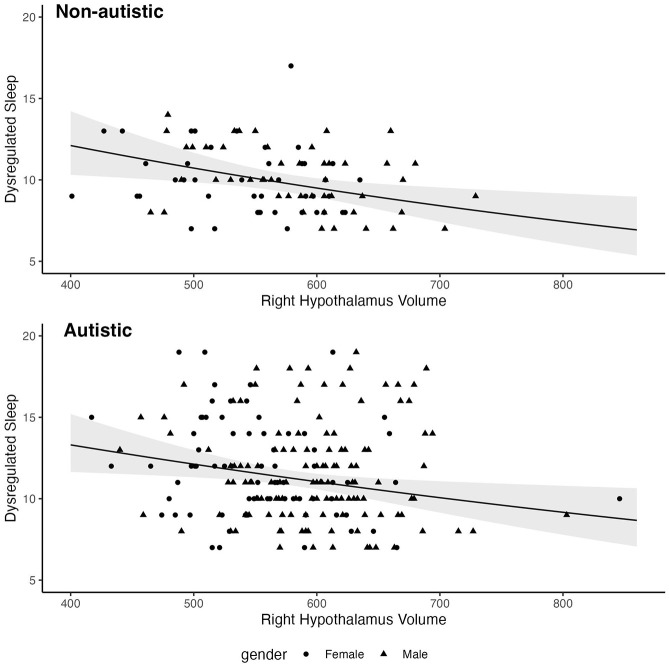
Associations between dysregulated sleep initiation/maintenance and right hypothalamus volume.

As presented in Table 2 and illustrated in Figure 2, the right hypothalamus showed a significant negative association with dysregulated sleep initiation/maintenance (*p* = 0.002, FDR adjusted *p* = 0.033). The diagnosis-by-right hypothalamus effect on dysregulated sleep initiation/maintenance was not significant (*p* = 0.99); that is, a similar association between right hypothalamus volume and dysregulated sleep initiation/maintenance was observed within both the autistic and non-autistic groups.

**Table 2.** Regional brain volume associations with sleep problem scores.

		ROI main effects			ROI-by-diagnosis effects		
ROI	Hemisphere	β	*SE*	*p* value^a^	β	*SE*	*p* value
Hypothalamus	L	0.17	0.09	0.674	–0.11	0.18	0.537
	R	–0.26	0.10	0.033	0.00	0.17	0.999
Hippocampus	L	–0.04	0.14	0.962	0.11	0.27	0.671
	R	0.04	0.13	0.962	–0.22	0.28	0.432
Thalamus	L	–0.39	0.25	0.868	0.33	0.40	0.414
	R	0.29	0.24	0.881	-0.37	0.40	0.357
Amygdala	L	–0.13	0.13	0.881	0.08	0.23	0.720
	R	0.19	0.14	0.868	–0.13	0.24	0.601
Nucleus Accumbens	L	0.01	0.12	0.942	0.10	0.23	0.660
	R	–0.05	0.12	0.881	–0.15	0.23	0.505
Pons	L	–0.04	0.09	0.942	0.03	0.14	0.844
	R	0.01	0.09	0.942	0.02	0.15	0.873
Caudate Nucleus	L	0.11	0.18	0.942	–0.31	0.41	0.445
	R	0.03	0.18	0.942	0.09	0.40	0.825
Globus Pallidus	L	0.10	0.13	0.881	0.13	0.26	0.634
	R	–0.02	0.14	0.881	–0.25	0.25	0.314
Putamen	L	0.14	0.25	0.942	–0.27	0.46	0.561
	R	–0.07	0.25	0.962	0.19	0.45	0.674

ROI = Region of interest; L = left hemisphere; R = right hemisphere; β = standardized regression coefficient; *SE* = standard error.

a *P* value for main effects is corrected for false discovery rate.

No other subcortical region showed a significant association with dysregulated sleep initiation/maintenance scores or significant ROI-by-diagnosis interactions (Table 2). Likelihood ratio tests showed no significant change to model fit when including three-way sex-by-diagnosis-by-ROI, or two-way sex-by-ROI, or sex-by-diagnosis terms (Supplemental Table S1). Thus, these interactions were not evaluated further. There were no significant main or interaction effects for regression models with the original CSHQ total score as the dependent variable (Supplemental Table S2).

### Mediation analysis

Given the significant association between right hypothalamus volume and dysregulated sleep initiation/maintenance, we conducted mediation analyses to examine the extent to which this association could be considered independent of externalizing and internalizing symptoms. Decreased right hypothalamus volume was associated with increased dysregulated sleep initiation/maintenance scores, which was partially mediated by externalizing symptoms (Table 3). Decreasing right hypothalamus volume was associated with increasing externalizing symptoms (*a* = –0.167), and increasing externalizing symptoms was associated with increasing dysregulated sleep initiation/maintenance scores (*b* = 0.339). A Monte Carlo 95% interval for the mediation effect (*ab* = –0.058) based on 2000 simulations was entirely below zero, 95% CI [–0.114, –0.008]. Although the effect of reduced right hypothalamus volume associated with increased dysregulated sleep initiation/maintenance reduced somewhat after accounting for externalizing symptoms, the association remained significant (*c′* = –0.207, *p* = 0.008). These effects did not significantly differ across autistic and non-autistic individuals (Supplemental Table S3). Analyses without the use of multiple imputation provided similar results (see Supplementary Methods). No significant effects of right hypothalamus were found for the model predicting internalizing symptoms (Supplemental Table S4); thus, no subsequent analyses were conducted to test for mediating effects of internalizing symptoms.

**Table 3.** Mediation analyses for externalizing symptoms.

	β	(*SE*)	95% CI	*p* value
*Direct effects*				
RH → Ext *(a)*	–0.167	(0.073)	[–0.317, –0.024]	0.022
Ext → Sleep Problems *(b)*	0.339	(0.063)	[0.216, 0.462]	<0.0001
RH → Sleep Problems *(c′)*	–0.207	(0.079)	[–0.362, –0.052]	0.008
*Mediation Effect*				
RH → Ext → Sleep Problems *(ab)*	–0.057	(0.028)	[–0.113, –0.008]	0.04

The model predicting Externalizing problems and the model predicting Sleep Problems also included Autism Diagnosis, Left Hypothalamus volume, Total volume, reported child sex, and age at scan as covariates. RH = Right Hypothalamus volume; Ext = Externalizing problems; β = Regression coefficient; SE = standard error.

## Discussion

This study examined associations between dysregulated sleep initiation/maintenance and volumes of nine subcortical brain regions implicated in regulating sleep (Mitra et al., 2016; Mizrahi-Kliger et al., 2018; Saper et al., 2010; Scammell et al., 2017; Yamashita & Yamanaka, 2017) in both autistic and non-autistic 2- to 4-year-olds. As hypothesized, reduced right hypothalamus volume was associated with increasingly dysregulated sleep initiation/maintenance in both autistic and non-autistic children; however, the magnitude of this association did not differ across diagnostic groups. Additional analyses found that externalizing (but not internalizing) symptoms partially accounted for the association between right hypothalamus volume and sleep initiation/maintenance dysregulation; again, the strength of these associations was similar across autistic and non-autistic individuals. No other subcortical regions, including the hippocampus, thalamus, pons, caudate nucleus, globus pallidus, and putamen, were associated with dysregulated sleep initiation/maintenance.

To our knowledge, this is the first neuroimaging study of children to examine the hypothalamus in association with dysregulated sleep initiation and maintenance. Our findings are consistent with other lines of research demonstrating that the hypothalamus, while small, includes several regions involved in distinct sleep regulating processes (Mizrahi-Kliger et al., 2018; Saper et al., 2010; Scammell et al., 2017; Yamashita & Yamanaka, 2017). This includes nuclei involved in regulating circadian rhythm (e.g. suprachiasmatic nucleus which is involved in melatonin production) as well as other regions (e.g. nuclei within the preoptic area) that regulate sleep in response to prior durations of wakefulness (Scammell et al., 2017). Also of interest, although we did not make any predictions relating to lateralization, we found that dysregulated sleep initiation/maintenance was associated with the right (and not left) hypothalamus. This is consistent with evidence that sleep regulation may be asymmetrical within the brain (Casagrande & Bertini, 2008; Kaufmann et al., 2006). For example, a study of adults that combined electroencephalography (EEG) and functional magnetic resonance imaging (fMRI) demonstrated that the greatest changes in brain activity when transitioning from wakefulness to sleep occurred in subcortical regions of the right hemisphere (Kaufmann et al., 2006); however, see Ding et al. (2021) for evidence of left hypothalamus involvement.

The finding that autistic and non-autistic groups did not differ in the strength of associations between right hypothalamus, dysregulated sleep initiation and maintenance, and externalizing symptoms is inconsistent with previous research demonstrating that autistic and non-autistic individuals do differ in physiological markers like melatonin (Melke et al., 2008) or circadian-relevant genes (Yang et al., 2016) that are integral to hypothalamic sleep regulating processes. Rather, the results of this study are more consistent with prior research demonstrating little difference between autistic and non-autistic individuals in neurobiological markers linked to sleep (Goldman et al., 2014; Niarchou et al., 2022).

This study builds upon several other neuroimaging studies investigating brain structure and sleep in children, albeit that none of these examined the hypothalamus. Studies on non-autistic school-age children have demonstrated that reduced volume of other subcortical regions are associated with greater sleep difficulties (Cheng et al., 2021; Hernandez et al., 2023; Riggins & Spencer, 2020). In contrast, a longitudinal study of infants, including those with a family history of autism, demonstrated that dysregulated sleep initiation at 6 and 12 months of age was associated with increased bilateral hippocampal volume at 6, 12, and 24 months of age, but only for those ultimately diagnosed with ASD at 24 months of age (MacDuffie et al., 2020). In contrast to the aforementioned neuroimaging studies of non-autistic school-age children and the study of autistic and non-autistic children across infancy and toddlerhood, we did not find dysregulated sleep to be associated with the volume of any other subcortical regions examined. One consideration relevant to interpreting this overall pattern of findings is differences across studies in sample ages, meaning that differences across studies may reflect that the degree sleep is associated with subcortical structures depends on developmental stage. Second, MacDuffie et al. (2020) employed a longitudinal approach while this study focused on concurrent associations between subcortical volume and dysregulated sleep initiation and maintenance. As such, results may reflect differences in timing between measurement of subcortical volume and dysregulated sleep initiation/maintenance. Given these methodological differences, there may be several plausible interpretations that integrate findings across studies. One potential interpretation is that while early dysregulation of sleep initiation/maintenance may be a marker for increasing hippocampal volume specific to autism, reduced right hypothalamic volume reflects a concurrent neurobiological mechanism for dysregulated sleep initiation and maintenance common to both autistic and non-autistic children. Finally, it is important to note that the ASD sample in the MacDuffie et al. (2020) study was identified through an infant-sibling design, whereas the ASD sample in this study was included based on confirmed autism diagnoses. Consequently, this study may have overlooked autistic children considered unlikely to be autistic or unable to obtain a formal diagnosis. Given these differences in ASD sample identification, an interpretation that cannot be ruled out is that associations between subcortical volume and sleep dysregulation might relate to different phenotypes within the autism spectrum.

Results from this study also extend our understanding concerning externalizing and internalizing symptoms co-occurring with dysregulated sleep for both autistic and non-autistic children (Abel et al., 2018; Cheng et al., 2021; Mannion & Leader, 2014; Shen et al., 2020). In particular, this study demonstrated that although externalizing symptoms were associated with reduced right hypothalamus volume and dysregulated sleep initiation/maintenance, they only partially accounted for the association between reduced right hypothalamus volume and increased dysregulated sleep initiation/maintenance across the full sample. This result parallels those of a recent study finding that ADHD symptoms partially mediated the association between dyssomnia and reduced volume across the insula, caudate, and putamen in a community sample of 7-year-olds (Shen et al., 2020). One interpretation of these findings is that while some reduced volume in brain regions is common to both dysregulated sleep initiation/maintenance and externalizing symptoms, additional variance in brain region volume is uniquely associated with dysregulated sleep initiation/maintenance. More specifically, findings from this study suggest that similar for both autistic and non-autistic children, some degree of reduction in gross hypothalamic volume contributes both to shared and unique aspects of externalizing symptoms and dysregulated sleep initiation/maintenance. Hence, although there may often be an assumption that mechanisms underlying difficulties with sleep are unique for specific populations, including autistic individuals, the findings from this study do not support this. Rather, the current findings are more consistent with the notion that difficulties initiating and maintaining sleep in autism share similar neurobiological mechanisms with such difficulties in typical development (Kamara & Beauchaine, 2020).

The findings of this study have implications for clinical practice supporting autistic and non-autistic individuals with sleep problems. First, findings linking the hypothalamus in sleep are in keeping with research demonstrating that sleep/wake regulation problems in autistic children may be treated with melatonin (Ballester et al., 2020), a hormone produced by the pineal gland which receives signals of light/dark cycles via the suprachiasmatic nucleus of the hypothalamus (MacLean & Malow, 2022). Syntheses of limited evidence from randomized controlled trials suggest that administration of exogenous melatonin in addition to behavioral approaches to managing sleep hygiene (e.g. establishing a regular bedtime routine, going to bed at a regular time, minimizing light and distractions in the bedroom) may be effective for treating sleep problems in some autistic children; however, further research is needed to address questions concerning such issues as long-term effects, dosage and timing, polypharmacy, and mechanisms of action (Cuomo et al., 2017; MacLean & Malow, 2022). Moreover, this study indicated that dysregulated sleep was associated with the hypothalamus directly, as well as externalizing symptoms, further highlighting that there may often be multiple targets for sleep interventions. Second, results of this study imply that dysregulated sleep for autistic children arises through at least some mechanisms that are similar to non-autistic children, consistent with evidence that sleep interventions developed for non-autistic children are also efficacious for autistic children (Cortese et al., 2020). Finally, underscoring the importance of careful consideration in the assessment of sleep, this study demonstrated that although a subset of CSHQ items reflecting dysregulated sleep initiation and maintenance was associated with right hypothalamus volume, the total CSHQ score was not. This emphasizes that total CSHQ scores provide a non-specific indication of sleep problems, and that the subset of items specific to initiating and maintaining sleep is more powerful to detect neurobiological underpinnings relating to these aspects of sleep.

This study was the first to investigate associations between subcortical brain volumes and sleep problems in autistic children near the age of diagnosis, and utilized a recently developed scale of dysregulated sleep initiation/maintenance that is equally reliable in both autistic and non-autistic individuals (Hatch et al., 2021). We used this scale because, to the best of our knowledge, it is the only parent-report measure of dysregulated sleep shown to be equally reliable across autistic and non-autistic children, which should be considered to assure valid groupwise comparisons of means and associations (Meredith, 1993). While these were strengths of the study, limitations should also be noted. First, sleep was measured only using a parent report. Although parent report measures of sleep, such as the one used in this study have unique strengths (e.g. reflecting sleep quality problems severe enough to be noticed by parents), objective methods such as video somnography and actigraphy are likely to capture variation in sleep parameters otherwise undetected (Gregory & Sadeh, 2016). Second, the spatial resolution of research standard MRI may be insufficient to distinguish between functionally distinct structures within some ROIs, including subregions of the hypothalamus. Thus, while subregions of the hypothalamus (tuberomammillary nucleus located in posterior hypothalamus, lateral hypothalamus, and suprachiasmatic nucleus within the ventral hypothalamus) are implicated in different sleep regulating processes (Mizrahi-Kliger et al., 2018; Saper et al., 2010; Scammell et al., 2017; Yamashita & Yamanaka, 2017), this study is unable to examine these regions specifically. Third, this study examined associations between subcortical volumes and sleep concurrently yet, as it is plausible that the strength of these relationships varies over time. Finally, while this study demonstrates smaller right hypothalamus is associated with dysregulated sleep initiation/maintenance in autistic and non-autistic children, this does not address why, on average, dysregulated sleep initiation and maintenance is elevated in autistic compared with non-autistic children. This may be due to several factors unique to autism, including sensory sensitivity (Manelis-Baram et al., 2022) and emotion dysregulation (Sivertsen et al., 2012), as well as the possibility that parent ratings on one domain (i.e. sleep) can be influenced by their perceptions in another domain (i.e. autism symptoms). Taken together, while this study provides further understanding of the neurobiological mechanisms underlying sleep difficulties in autism, a more complete understanding likely requires longitudinal research utilizing multiple methods of measuring both sleep and markers of underlying neurobiological processes.

In conclusion, this study investigated associations between subcortical brain volumes and dysregulated sleep initiation and maintenance in 2- to 4-year-old autistic and non-autistic children. Results suggest that across 2 to 4 years of age, reduced right hypothalamus volume is significantly associated with increasingly dysregulated sleep initiation and maintenance. Although autistic children experience greater dysregulation of sleep initiation and maintenance compared with non-autistic peers, autistic and non-autistic children do not significantly differ in the extent to which dysregulated sleep initiation/maintenance is associated with reduced hypothalamus volume, nor the extent to which this association is partially accounted for by externalizing (but not internalizing) problems.

## Supplemental Material

sj-docx-1-aut-10.1177_13623613251352249 – Supplemental material for Hypothalamic volume is associated with dysregulated sleep in autistic and non-autistic young children Supplemental material, sj-docx-1-aut-10.1177_13623613251352249 for Hypothalamic volume is associated with dysregulated sleep in autistic and non-autistic young children by Burt Hatch, Derek Sayre Andrews, Brett Dufour, Shayan M Alavynejad, Joshua K Lee, Sally Rogers, Marjorie Solomon, Meghan Miller and Christine Wu Nordahl in Autism

sj-docx-2-aut-10.1177_13623613251352249 – Supplemental material for Hypothalamic volume is associated with dysregulated sleep in autistic and non-autistic young children Supplemental material, sj-docx-2-aut-10.1177_13623613251352249 for Hypothalamic volume is associated with dysregulated sleep in autistic and non-autistic young children by Burt Hatch, Derek Sayre Andrews, Brett Dufour, Shayan M Alavynejad, Joshua K Lee, Sally Rogers, Marjorie Solomon, Meghan Miller and Christine Wu Nordahl in Autism

sj-docx-3-aut-10.1177_13623613251352249 – Supplemental material for Hypothalamic volume is associated with dysregulated sleep in autistic and non-autistic young children Supplemental material, sj-docx-3-aut-10.1177_13623613251352249 for Hypothalamic volume is associated with dysregulated sleep in autistic and non-autistic young children by Burt Hatch, Derek Sayre Andrews, Brett Dufour, Shayan M Alavynejad, Joshua K Lee, Sally Rogers, Marjorie Solomon, Meghan Miller and Christine Wu Nordahl in Autism

sj-docx-4-aut-10.1177_13623613251352249 – Supplemental material for Hypothalamic volume is associated with dysregulated sleep in autistic and non-autistic young children Supplemental material, sj-docx-4-aut-10.1177_13623613251352249 for Hypothalamic volume is associated with dysregulated sleep in autistic and non-autistic young children by Burt Hatch, Derek Sayre Andrews, Brett Dufour, Shayan M Alavynejad, Joshua K Lee, Sally Rogers, Marjorie Solomon, Meghan Miller and Christine Wu Nordahl in Autism

sj-docx-5-aut-10.1177_13623613251352249 – Supplemental material for Hypothalamic volume is associated with dysregulated sleep in autistic and non-autistic young children Supplemental material, sj-docx-5-aut-10.1177_13623613251352249 for Hypothalamic volume is associated with dysregulated sleep in autistic and non-autistic young children by Burt Hatch, Derek Sayre Andrews, Brett Dufour, Shayan M Alavynejad, Joshua K Lee, Sally Rogers, Marjorie Solomon, Meghan Miller and Christine Wu Nordahl in Autism
