# Supplementary material for: Hypothalamic volume is associated with dysregulated sleep in autistic and non-autistic young children
Source: Autism. 2025 Jul 9;29(11):2885–97. doi: 10.1177/13623613251352249 (PMC12531386; doi:10.1177/13623613251352249)
Supplement: sj-docx-1-aut-10.1177_13623613251352249 – Supplemental material for Hypothalamic volume is associated with dysregulated sleep in autistic and non-autistic young children [file sj-docx-1-aut-10.1177_13623613251352249.docx]

**SUPPLEMENTARY METHODS AND RESULTS**

*Participants*

Participants were required to be English speakers, ambulatory, have no contraindications for MRI, no suspected vision or hearing problems, and no known genetic disorders or neurological conditions. ASD diagnosis was confirmed by research reliable clinical psychologists using the Autism Diagnostic Observation Schedule-Generic (ADOS-G; Lord et al., 2000) or ADOS-2, (Lord et al., 2012) the Autism Diagnostic Interview-Revised (ADI-R: Rutter, Le Couteur, et al., 2003), and application of *DSM-IV-TR*(American Psychiatric Association, 2000) or *DSM-5* criteria (American Psychiatric Association, 2013). All autistic participants met ADOS-2 cutoff scores for either autism or ASD, exceeded ADI-R cutoff scores for autism on either the social or communication subscale and were within two points of this criterion on the other subscale, *and* met *DSM-IV* criteria for Autistic Disorder, Asperger's Disorder, or Pervasive Developmental Disorder Not Otherwise Specified, *or* *DSM-5* criteria for ASD. ADOS calibrated severity scores were calculated to allow comparison of autism symptom severity across participants tested with different ADOS modules (Gotham et al., 2012).

At study entry, non-autistic control participants were screened for autism traits using the Social Communication Questionnaire (SCQ; i.e. scores below 11; Rutter, Bailey, et al., 2003) and were required to have no first-degree relatives with an ASD diagnosis. The Mullen Scales of Early Learning (MSEL: Mullen, 1995) was used to assess for developmental delays. Non-autistic control participants were excluded if their MSEL Early Learning Composite score fell below two standard deviations from the mean. One non-autistic male was assessed using the Stanford-Binet abbreviated battery measure of IQ instead of the MSEL and is included in the sample (Roid, 2003).

Autistic and non-autistic participants did not significantly differ in age at assessment (*t*=-0.94, *p*=0.34). The autistic group contained a slightly larger proportion of males to females, with a chi-square test of this ratio nearing significance (χ^2^=3.38, *p*=0.06)*.* Compared to the non-autistic group, autistic participants had significantly lower Early Learning Composite scores (*t*=19.93, *p*<0.001).

*Magnetic Resonance Imaging Acquisition and Region of Interest Approach*

MRI scans were acquired during natural nocturnal sleep (Mori et al., 2016) at UC Davis Research Imaging Center from 2006-2019 on a 3T Siemens Trio whole-body MRI system using an eight-channel head coil. High resolution, T1-weighted structural scans were acquired using a 3-dimensional magnetization-prepared rapid acquisition gradient-echo (MPRAGE) sequence (TR 2,170 milliseconds; TE 4.86 milliseconds; matrix 256 × 256; 192 slices in the sagittal direction; 1.0-mm isotropic voxels). Image distortion associated with changes in hardware and software over time was controlled for by scanning a calibration phantom (ADNI MAGPHAM, The Phantom Laboratory) at the end of each MRI session and applying distortion correction to each MPRAGE image (Image Owl, Inc, Greenwich, NY; <http://www.imageowl.com/>).

Distortion corrected, anonymized, and defaced MPRAGE images were uploaded to MRICloud ([https://mricloud.org](https://mricloud.org/)) (Wang et al., 2013) and segmented into 289 anatomically defined regions using a multi-atlas approach in the fully automated MRICloud T1-Segmentation pipeline v7A (Mori et al., 2016; Wang et al., 2013). An age-specific atlas was used for each individual participant according to their age in years at image acquisition. For 2- and 3-year-olds, the multi-atlas sets (each comprising 13 pediatric atlases) were optimized using data from each age group and are freely available on MRI Cloud (UC Davis 2 Years and UC Davis 3 Years). For children 4 years and older, the Pediatric 4-8 Years multi-atlas (based on 10 pediatric atlases) from the Johns Hopkins University inventory was utilized. Whole-brain segmentation output for each participant was downloaded and visually inspected for segmentation quality. Total hemispheric volumes were extracted and summed to calculate total cerebral volume (TCV). Volumes were extracted and exported for further statistical analysis from nine a priori regions of interest (Mitra et al., 2016; Mizrahi-Kliger et al., 2018; Saper et al., 2010; Scammell et al., 2017): the left and right hypothalamus, thalamus, amygdala, caudate nucleus, putamen, hippocampus, nucleus accumbens, globus pallidus, and pons (illustrated in Figure 1).

From the entire recruited sample, participants who did and didn’t successfully complete MRI scans did not significantly differ in CSHQ total (t = 0.32, *p* = .748), dysregulated sleep initiation/maintenance (t = 0.72, *p* = .471), externalizing (t = 1.62, *p* = .104) or internalizing symptoms (t = 1.60, *p* = .110). There was no significant effect for scan status-by-diagnosis interactions for CSHQ Total (*F =* 0.64, *p* = .424), dysregulated sleep initiation/maintenance (*F =* 1.37, *p* = .243), externalizing (*F =* 0.08, *p* = .790) or internalizing symptoms (*F =* 0.05, *p* = .822). This suggests failure to complete MRI was not associated with differences in sleep measures across the entire sample, for autistic and non-autistic participants.

*Multiple imputation*

The percentage of missing values for the CBCL six sub-scales that comprise the externalizing and internalizing scales varied between 4 and 6%. Twenty-eight out of 295 participants (9%) had incomplete data on CBCL sub-scales that comprise the externalizing and internalizing scales. For the total sample missing data was associated with lower DQ scores. For the ASD group missing data was associated with lower DQ scores and higher SCQ scores. We conducted multiple imputation by chained equations to create and analyze 200 multiply imputed datasets.

Incomplete sub-scales that comprise the externalizing and internalizing scales of the CBCL were imputed fully conditional on mediation model variables as well as the DQ and SCQ variables associated with missingness. Given that we tested if mediation was moderated by autism group multiple imputation was conducted separately by autism group status. Imputation was conducted using classification and regression trees using the ‘mice’ (version 3.15) package (van Buuren & Groothuis-Oudshoorn, 2011). For comparison, we also performed the analysis on the subset of complete cases (Tables S5 and S6).

**SUPPLEMENT TABLES**

Table S1: Likelihood Ratio Testing of Interaction Terms

|  | Sex-by-Diagnosis-by-ROI | Sex-by-ROI | Sex-by-Diagnosis |
| --- | --- | --- | --- |
| **ROI** | **p - value** | **p - value** | **p - value** |
| Hypothalamus | 0.434 | 0.140 | 0.140 |
| Hippocampus | 0.869 | 0.793 | 0.793 |
| Thalamus | 0.833 | 0.324 | 0.324 |
| Amygdala | 0.939 | 0.858 | 0.858 |
| Nucleus Accumbens | 0.508 | 0.327 | 0.327 |
| Globus Pallidus | 0.197 | 0.445 | 0.230 |
| Putamen | 0.780 | 0.663 | 0.321 |
| Pons | 0.446 | 0.661 | 0.504 |
| Caudate Nucleus | 0.813 | 0.548 | 0.314 |

Notes: ROI = region of interest

Table S2: Regional Brain Volume Associations with CSHQ Total score

|  | | ROI main effects | | |  | ROI - by - Diagnosis effects | | |
| --- | --- | --- | --- | --- | --- | --- | --- | --- |
| **ROI** | **Hemisphere** | **b** | **SE** | **P value** |  | **b** | **SE** | **P value** |
| Hypothalamus | L | 0.16 | 0.10 | 0.537 |  | -0.17 | 0.18 | 0.334 |
|  | R | -0.16 | 0.10 | 0.999 |  | 0.04 | 0.17 | 0.832 |
| Hippocampus | L | 0.12 | 0.14 | 0.671 |  | -0.06 | 0.27 | 0.832 |
|  | R | -0.08 | 0.13 | 0.432 |  | -0.04 | 0.29 | 0.891 |
| Thalamus | L | -0.48 | 0.25 | 0.414 |  | 0.56 | 0.41 | 0.171 |
|  | R | 0.32 | 0.25 | 0.357 |  | -0.56 | 0.41 | 0.169 |
| Amygdala | L | -0.07 | 0.13 | 0.720 |  | 0.13 | 0.24 | 0.589 |
|  | R | 0.02 | 0.14 | 0.601 |  | -0.16 | 0.25 | 0.524 |
| Nucleus Accumbens | L | 0.00 | 0.12 | 0.660 |  | 0.12 | 0.24 | 0.620 |
|  | R | 0.01 | 0.12 | 0.505 |  | -0.19 | 0.23 | 0.408 |
| Pons | L | -0.05 | 0.09 | 0.844 |  | 0.16 | 0.15 | 0.264 |
|  | R | 0.00 | 0.09 | 0.873 |  | -0.01 | 0.15 | 0.964 |
| Caudate Nucleus | L | 0.19 | 0.19 | 0.445 |  | -0.32 | 0.41 | 0.437 |
|  | R | -0.10 | 0.19 | 0.825 |  | 0.18 | 0.41 | 0.656 |
| Globus Pallidus | L | 0.08 | 0.13 | 0.634 |  | 0.19 | 0.27 | 0.483 |
|  | R | -0.08 | 0.14 | 0.314 |  | -0.22 | 0.26 | 0.388 |
| Putamen | L | -0.10 | 0.25 | 0.561 |  | 0.02 | 0.47 | 0.966 |
|  | R | 0.19 | 0.25 | 0.674 |  | -0.07 | 0.46 | 0.871 |

Notes: ROI = Region of Interest; L = left hemisphere; R = right hemisphere; **b** = Standardized regression coefficient; SE = Standard Error.

Table S3: Moderating effects for autism diagnosis status on direct and mediating effects

| **Effect** | **χ^2^** | **p - value** |
| --- | --- | --- |
| ***Direct effects*** |  |  |
| RH 🡪 Ext | 0.442 | 0.506 |
| Ext 🡪 Sleep Problems | 1.194 | 0.275 |
| RH 🡪 Sleep Problems | 0.082 | 0.774 |
| ***Mediation Effect*** |  |  |
| R.H. -> Ext. -> Sleep Problems | 0.057 | 0.811 |

Note: RH = Right Hypothalamus volume. Ext = Externalizing problems. The model predicting Externalizing problems and the model predicting Sleep Problems also included Left Hypothalamus volume, Total volume, reported child sex, and age at scan as covariates. Each model was estimated using a structural equation framework that allowed for effects to be freely estimated for the ASD and Non-ASD group. The χ^2^ statistic is a likelihood ratio test indicating if the effect freely estimated across the ASD and Non-ASD is significantly different from the effect being assumed equal across the ASD and Non-ASD groups.

Table S4: Regression analyses for internalizing symptoms

|  | **β** | **SE** | **t-value** | **p - value** |
| --- | --- | --- | --- | --- |
| Intercept | 0.98 | 0.34 | 2.91 | 0.004 |
| Autism Diagnosis | 1.38 | 0.10 | 13.62 | <0.001 |
| Right Hypothalamus | 0.06 | 0.07 | 0.83 | 0.405 |
| Left Hypothalamus | -0.05 | 0.07 | -0.80 | 0.422 |
| Sex | -0.08 | 0.10 | -0.80 | 0.426 |
| Age | -0.01 | 0.01 | -1.58 | 0.116 |
| Total Cerebral Volume | -0.07 | 0.06 | -1.05 | 0.295 |

Notes: **b** = Regression coefficient. SE = Standard Error

Table S5: Moderating effects for autism diagnosis status on direct and mediating effects from analyses conducted without multiple imputation

| **Effect** | **χ^2^** | **p - value** |
| --- | --- | --- |
| ***Direct effects*** |  |  |
| RH 🡪 Ext | 0.346 | 0.556 |
| Ext 🡪 Sleep Problems | 0.824 | 0.363 |
| RH 🡪 Sleep Problems | 0.825 | 0.364 |
| ***Mediation Effect*** |  |  |
| R.H. -> Ext. -> Sleep Problems | 0.067 | 0.796 |

Note: RH = Right Hypothalamus volume. Ext = Externalizing problems. The model predicting Externalizing problems and the model predicting Sleep Problems also included Left Hypothalamus volume, Total volume, reported child sex, and age at scan as covariates. Each model was estimated using a structural equation framework that allowed for effects to be freely estimated for the ASD and Non-ASD group. The χ^2^ statistic is a likelihood ratio test indicating if the effect freely estimated across the ASD and Non-ASD is significantly different from the effect being assumed equal across the ASD and Non-ASD groups.

Table S6. Mediation analyses for externalizing symptoms conducted without multiple imputation

|  | **β** | **(SE)** | **95% CI** | **p - value** |
| --- | --- | --- | --- | --- |
| ***Direct effects*** | | | | |
| RH 🡪 Ext *(a)* | -0.161 | 0.073 | -0.313, -0.012 | 0.028 |
| Ext 🡪 Sleep Problems *(b)* | 0.365 | 0.063 | 0.242, 0.489 | < .001 |
| RH 🡪 Sleep Problems *(c’)* | -0.211 | 0.077 | -0.363, -0.059 | 0.007 |
| ***Mediation Effect*** |  |  |  |  |
| RH 🡪 Ext 🡪 Sleep Problems *(ab)* | -0.059 | 0.029 | -0.115, -0.003 | 0.040 |

**SUPPLEMENT SECTION REFERENCES**

American Psychiatric Association. (2000). *Diagnostic and statistical manual of mental disorders* (4th, text revision ed.). American Psychiatric Association.

American Psychiatric Association. (2013). *Diagnostic and statistical manual of mental disorders* (5th ed.). American Psychiatric Association.

Gotham, K., Pickles, A., & Lord, C. (2012). Trajectories of Autism Severity in Children Using Standardized ADOS Scores. *Pediatrics*, *130*(5), e1278–e1284. https://doi.org/10.1542/peds.2011-3668

Lord, C., Risi, S., Lambrecht, L., Cook, Jr., E. H., Leventhal, B. L., DiLavore, P. C., Pickles, A., & Rutter, M. (2000). The Autism Diagnostic Observation Schedule—Generic: A standard measure of social and communication deficits associated with the spectrum of autism. *Journal of Autism and Developmental Disorders*, *30*(3), 205–223. https://doi.org/10.1023/A:1005592401947

Lord, C., Rutter, M., DiLavore, P. C., Gotham, K., & Bishop, S. (2012). *Autism diagnostic observation schedule* (2nd ed.). western Psychological Services.

Mitra, A., Snyder, A. Z., Hacker, C. D., Pahwa, M., Tagliazucchi, E., Laufs, H., Leuthardt, E. C., & Raichle, M. E. (2016). Human cortical–hippocampal dialogue in wake and slow-wave sleep. *Proceedings of the National Academy of Sciences*, *113*(44), E6868–E6876. https://doi.org/10.1073/pnas.1607289113

Mizrahi-Kliger, A. D., Kaplan, A., Israel, Z., & Bergman, H. (2018). Desynchronization of slow oscillations in the basal ganglia during natural sleep. *Proceedings of the National Academy of Sciences of the United States of America*, *115*(18), E4274–E4283. https://doi.org/10.1073/pnas.1720795115

Mori, S., Wu, D., Ceritoglu, C., Li, Y., Kolasny, A., Vaillant, M. A., Faria, A. V., Oishi, K., & Miller, M. I. (2016). MRICloud: Delivering High-Throughput MRI Neuroinformatics as Cloud-Based Software as a Service. *Computing in Science & Engineering*, *18*(5), 21–35. https://doi.org/10.1109/MCSE.2016.93

Mullen, E. M. (1995). *Mullen Scales of Early Learning, AGS Edition: Manual and Item Administrative Books*. American Guidance Services, Inc.

Roid, G. H. (2003). *Stanford Binet intelligence scales (5th ed.)*. Riverside Publishing.

Rutter, M., Bailey, A. J., & Lord, C. (2003). *Social Communication Questionnaire (SCQ).* Western Psychological Services.

Rutter, M., Le Couteur, A., & Lord, C. (2003). *Autism Diagnostic Interview—Revised* (Vol. 29). Western Psychological Services.

Saper, C. B., Fuller, P. M., Pedersen, N. P., Lu, J., & Scammell, T. E. (2010). Sleep state switching. *Neuron*, *68*(6), 1023–1042. https://doi.org/10.1016/j.neuron.2010.11.032

Scammell, T. E., Arrigoni, E., & Lipton, J. O. (2017). Neural circuitry of wakefulness and sleep. *Neuron*, *93*(4), 747–765. https://doi.org/10.1016/j.neuron.2017.01.014

van Buuren, S., & Groothuis-Oudshoorn, K. (2011). mice: Multivariate Imputation by Chained Equations in R. *Journal of Statistical Software*, *45*(3). https://doi.org/10.18637/jss.v045.i03

Wang, H., Suh, J. W., Das, S. R., Pluta, J. B., Craige, C., & Yushkevich, P. A. (2013). Multi-Atlas Segmentation with Joint Label Fusion. *IEEE Transactions on Pattern Analysis and Machine Intelligence*, *35*(3), 611–623. https://doi.org/10.1109/TPAMI.2012.143
