# Supplementary material for: Hypothalamic volume is associated with dysregulated sleep in autistic and non-autistic young children
Source: Autism. 2025 Jul 9;29(11):2885–97. doi: 10.1177/13623613251352249 (PMC12531386; doi:10.1177/13623613251352249)
Supplement: sj-docx-2-aut-10.1177_13623613251352249 – Supplemental material for Hypothalamic volume is associated with dysregulated sleep in autistic and non-autistic young children [file sj-docx-2-aut-10.1177_13623613251352249.docx]

CSHQ total score cleaned

Burt

2025-04-30

# Load required libraries
library(rio) # For data import/export

library(lavaan) # For structural equation modeling

library(semTools) # SEM tools

library(tidyverse) # For data manipulation and visualization

library(mice) # For multiple imputation

library(psych) # Various statistical functions

library(mitml) # Multiple imputation tools

library(miceadds) # Additional tools for 'mice'

library(car) # Companion to Applied Regression

library(readxl) # For reading Excel files

# Load raw data from CSV
df <- read.csv("MORI_raw.df_061920.csv")

# Load additional dataset and process it
dataset_2020_04_30 <- read_excel("dataset_2020-04-30.xlsx") %>%
 filter(visit == 1) %>% # Filter for first visit
 select(subj_id, demo_ch_rep_eth, demo_ch_rep_race, demo_ann_in) %>% # Select relevant columns
 mutate(
 # Create an ordered income category variable
 Income_ord = case_when(
 demo_ann_in %in% c("Under $10 000", "$10 000-$29 000", "$30 000-$49 000", "$50 000-$74 999", "$75 000-$99 999") ~ "Less than 100,000",
 demo_ann_in == "$100 000-$149 999" ~ "$100 000-$149 999",
 demo_ann_in == "$150 000 and above" ~ "$150 000 and above"
 ),
 Income_ord.n = as.ordered(case_when(
 demo_ann_in %in% c("Under $10 000", "$10 000-$29 000", "$30 000-$49 000", "$50 000-$74 999", "$75 000-$99 999") ~ 0,
 demo_ann_in == "$100 000-$149 999" ~ 1,
 demo_ann_in == "$150 000 and above" ~ 2
 )),
 # Handle missing race and ethnicity values
 demo_ch_rep_race = replace_na(demo_ch_rep_race, "Not reported"),
 demo_ch_rep_eth = replace_na(demo_ch_rep_eth, "Not reported"),
 # Group race into broader categories
 Race = case_when(
 demo_ch_rep_race == "African American/Black" ~ "African American/Black",
 demo_ch_rep_race == "Asian" ~ "Asian",
 demo_ch_rep_race %in% c("Mixed", "Other") ~ "Mixed/Other",
 demo_ch_rep_race %in% c("Not reported", "Refused") ~ "Refused/Not reported",
 demo_ch_rep_race == "White/Caucasian" ~ "White/Caucasian"
 )
 )

# Merge the processed dataset with the main data by subject ID
df <- dplyr::left_join(df, dataset_2020_04_30, by = "subj_id")

# Display column names of the processed dataset
names(dataset_2020_04_30)

# Specify variable formats
df$subj_id <- factor(df$subj_id)
df$gender <- factor(df$gender)
df$app_diagnosis <- factor(df$app_diagnosis)
df$ados_ccs <- as.numeric(df$ados_ccs)

# Select a subset of variables for analysis
df.sub.1 <- dplyr::select(
 df,
 subj_id, demo_ch_rep_eth, demo_ch_rep_race, demo_ann_in, Income_ord, Income_ord.n, Race,
 gender, app_diagnosis, scan_age, msel_dq,
 mori_total_volume, hippo_l, hippo_r, hypothalamus_l, hypothalamus_r,
 thalamus_l, thalamus_r, amyg_l, amyg_r, nucaccumbens_l, nucaccumbens_r,
 gp_l, gp_r, put_l, put_r, pons_l, pons_r, caud_l, caud_r,
 SLEEP_1:SLEEP_48, cshq_sum, cshq_total,
 cbcl_emotionally_reactive_raw, cbcl_anxious_depressed_raw,
 cbcl_somatic_complaints_raw, cbcl_withdrawn_raw, cbcl_sleep_problem_raw,
 cbcl_attention_problem_raw, cbcl_aggressive_behavior_raw, cbcl_externalizing_raw,
 cbcl_internalizing_raw, cbcl_externalizing_t, cbcl_internalizing_t, ados_ccs
)


# Standardize and transform variables for regression models
df.sub.1$cshq_total.sd <- scale(log(df.sub.1$cshq_total), center = TRUE, scale = TRUE)
df.sub.1 <- df.sub.1 %>%
 mutate(across(
 c(hypothalamus_l, hypothalamus_r, hippo_l, hippo_r, thalamus_l, thalamus_r,
 amyg_l, amyg_r, nucaccumbens_l, nucaccumbens_r, gp_l, gp_r, put_l, put_r,
 pons_l, pons_r, caud_l, caud_r),
 ~ scale(.x, center = TRUE, scale = TRUE),
 .names = "{col}.sd"
 ))

# Run multiple linear regression models for each brain region
library(broom)

lmhypothallog <- lm(cshq_total.sd ~ gender + scan_age + mori_total_volume + app_diagnosis * (hypothalamus_l.sd + hypothalamus_r.sd),df.sub.1)
hypothalanova <- Anova(lmhypothallog,type = "II")


lmhippolog <- lm(cshq_total.sd ~ gender + scan_age + mori_total_volume + app_diagnosis * (hippo_l.sd + hippo_r.sd),df.sub.1)
hippoanova <- Anova(lmhippolog,type = "II")

lmthalamuslog <- lm(cshq_total.sd ~ gender + scan_age + mori_total_volume + app_diagnosis * (thalamus_l.sd + thalamus_r.sd),df.sub.1)
thalamusanova <- Anova(lmthalamuslog,type = "II")

lmamyglog <- lm(cshq_total.sd ~ gender + scan_age + mori_total_volume + app_diagnosis * (amyg_l.sd + amyg_r.sd),df.sub.1)
amyganova <- Anova(lmamyglog,type = "II")

lmnucaccumbenslog <- lm(cshq_total.sd ~ gender + scan_age + mori_total_volume + app_diagnosis * (nucaccumbens_l.sd + nucaccumbens_r.sd),df.sub.1)
nucaccumbensanova <- Anova(lmnucaccumbenslog,type = "II")

lmponslog <- lm(cshq_total.sd ~ gender + scan_age + mori_total_volume + app_diagnosis * (pons_l.sd + pons_r.sd),df.sub.1)
ponsanova <- Anova(lmponslog,type = "II")

lmcaudlog <- lm(cshq_total.sd ~ gender + scan_age + mori_total_volume + app_diagnosis * (caud_l.sd + caud_r.sd),df.sub.1)
caudanova <- Anova(lmcaudlog,type = "II")

lmgplog <- lm(cshq_total.sd ~ gender + scan_age + mori_total_volume + app_diagnosis * (gp_l.sd + gp_r.sd),df.sub.1)
gpanova <- Anova(lmgplog,type = "II")

lmputlog <- lm(cshq_total.sd ~ gender + scan_age + mori_total_volume + app_diagnosis * (put_l.sd + put_r.sd),df.sub.1)
putanova <- Anova(lmputlog,type = "II")

# Create a list of regression models for different brain regions
# Each model corresponds to a brain region and is stored with a descriptive name
models <- list(
 hypothalamus = lmhypothallog, # Model for hypothalamus
 hippocampus = lmhippolog, # Model for hippocampus
 thalamus = lmthalamuslog, # Model for thalamus
 amygdala = lmamyglog, # Model for amygdala
 nucaccumbens = lmnucaccumbenslog, # Model for nucleus accumbens
 pons = lmponslog, # Model for pons
 caudate = lmcaudlog, # Model for caudate
 gp = lmgplog, # Model for globus pallidus
 putamen = lmputlog # Model for putamen
)

# Extract regression coefficients (betas) and standard errors for main effects
# This focuses on the main effects of the brain regions (assumed to be 6th and 7th terms in each model)
main_results <- lapply(names(models), function(region_name) {
 tidy_mod <- broom::tidy(models[[region_name]]) %>%
 select(term, estimate, std.error) # Keep only the term, estimate, and std.error columns
 tidy_mod[c(6, 7), ] # Extract rows corresponding to main effects of the brain region
})

# Combine the results into a single data frame
df <- as.data.frame(do.call(rbind, main_results))

# Add an ID column to the data frame to maintain the order of terms
df$id <- 1:nrow(df)

# Extract p-values for main effects from the ANOVA results
# Note: Ensure the ANOVA objects (e.g., hypothalanova, hippoanova) are available in your environment
P_CSHQ_total <- data.frame(
 "hypothalamus_l.sd" = hypothalanova$`Pr(>F)`[5],
 "hypothalamus_r.sd" = hypothalanova$`Pr(>F)`[6],
 "hippo_l.sd" = hippoanova$`Pr(>F)`[5],
 "hippo_r.sd" = hippoanova$`Pr(>F)`[6],
 "thalamus_l.sd" = thalamusanova$`Pr(>F)`[5],
 "thalamus_r.sd" = thalamusanova$`Pr(>F)`[6],
 "amyg_l.sd" = amyganova$`Pr(>F)`[5],
 "amyg_r.sd" = amyganova$`Pr(>F)`[6],
 "nucaccumbens_l.sd" = nucaccumbensanova$`Pr(>F)`[5],
 "nucaccumbens_r.sd" = nucaccumbensanova$`Pr(>F)`[6],
 "pons_l.sd" = ponsanova$`Pr(>F)`[5],
 "pons_r.sd" = ponsanova$`Pr(>F)`[6],
 "caud_l.sd" = caudanova$`Pr(>F)`[5],
 "caud_r.sd" = caudanova$`Pr(>F)`[6],
 "gp_l.sd" = gpanova$`Pr(>F)`[5],
 "gp_r.sd" = gpanova$`Pr(>F)`[6],
 "put_l.sd" = putanova$`Pr(>F)`[5],
 "put_r.sd" = putanova$`Pr(>F)`[6]
)

# Extract regression coefficients (betas) and standard errors for main effects
# This focuses on the main effects of the brain regions (assumed to be 6th and 7th terms in each model)
main_results <- lapply(names(models), function(region_name) {
 tidy_mod <- broom::tidy(models[[region_name]]) %>%
 select(term, estimate, std.error) # Keep only the term, estimate, and std.error columns
 tidy_mod[c(6, 7), ] # Extract rows corresponding to main effects of the brain region
})

# Combine the results into a single data frame
df <- as.data.frame(do.call(rbind, main_results))

# Add an ID column to the data frame to maintain the order of terms
df$id <- 1:nrow(df)

# Extract p-values for main effects from the ANOVA results
# Note: Ensure the ANOVA objects (e.g., hypothalanova, hippoanova) are available in your environment
P_CSHQ_total <- data.frame(
 "hypothalamus_l.sd" = hypothalanova$`Pr(>F)`[5],
 "hypothalamus_r.sd" = hypothalanova$`Pr(>F)`[6],
 "hippo_l.sd" = hippoanova$`Pr(>F)`[5],
 "hippo_r.sd" = hippoanova$`Pr(>F)`[6],
 "thalamus_l.sd" = thalamusanova$`Pr(>F)`[5],
 "thalamus_r.sd" = thalamusanova$`Pr(>F)`[6],
 "amyg_l.sd" = amyganova$`Pr(>F)`[5],
 "amyg_r.sd" = amyganova$`Pr(>F)`[6],
 "nucaccumbens_l.sd" = nucaccumbensanova$`Pr(>F)`[5],
 "nucaccumbens_r.sd" = nucaccumbensanova$`Pr(>F)`[6],
 "pons_l.sd" = ponsanova$`Pr(>F)`[5],
 "pons_r.sd" = ponsanova$`Pr(>F)`[6],
 "caud_l.sd" = caudanova$`Pr(>F)`[5],
 "caud_r.sd" = caudanova$`Pr(>F)`[6],
 "gp_l.sd" = gpanova$`Pr(>F)`[5],
 "gp_r.sd" = gpanova$`Pr(>F)`[6],
 "put_l.sd" = putanova$`Pr(>F)`[5],
 "put_r.sd" = putanova$`Pr(>F)`[6]
)

P_CSHQ_total <- P_CSHQ_total %>%
 # Convert wide format to long format
 pivot_longer(
 cols = everything(),
 names_to = "term",
 values_to = "p_value"
 )

table_s2_fx <- merge(df, P_CSHQ_total, by = "term")
table_s2_fx <- table_s2_fx[order(table_s2_fx$id), ] %>%
 # Create new columns
 mutate(
 # Remove .sd suffix and extract hemisphere
 hemisphere = ifelse(str_detect(term, "_l\\.sd"), "left", "right"),
 # Clean region names by removing hemisphere suffixes
 region = str_remove(term, "_[lr]\\.sd$")
 ) %>%
 # Reorder columns
 select(region, hemisphere, estimate, std.error, p_value) %>%
 mutate_at(vars(estimate, std.error), funs(round(., 2))) %>%
 mutate_at(vars(p_value), funs(round(., 3)))

#table_s2_fx

# Create summary table for interaction terms

main_results_int <- lapply(names(models), function(region_name) {
 tidy_mod <- broom::tidy(models[[region_name]]) %>%
 select(term, estimate, std.error)
 tidy_mod[c(8,9),]
})

df_interactions <- as.data.frame(do.call(rbind, main_results_int))


df_interactions$term <- str_remove(df_interactions$term , "app_diagnosisTD:")

df_interactions$id <- 1:nrow(df_interactions)


P_CSHQ_total_INTERACTION <-data.frame("hypothalamus_l.sd"=hypothalanova$`Pr(>F)`[7],"hypothalamus_r.sd"=hypothalanova$`Pr(>F)`[8],"hippo_l.sd"=hippoanova$`Pr(>F)`[7],"hippo_r.sd"= hippoanova$`Pr(>F)`[8],"thalamus_l.sd"= thalamusanova$`Pr(>F)`[7],"thalamus_r.sd"= thalamusanova$`Pr(>F)`[8],"amyg_l.sd"= amyganova$`Pr(>F)`[7],"amyg_r.sd"= amyganova$`Pr(>F)`[8],"nucaccumbens_l.sd"= nucaccumbensanova$`Pr(>F)`[7],"nucaccumbens_r.sd"= nucaccumbensanova$`Pr(>F)`[8],"pons_l.sd"= ponsanova$`Pr(>F)`[7],"pons_r.sd"= ponsanova$`Pr(>F)`[8],"caud_l.sd"= caudanova$`Pr(>F)`[7],"caud_r.sd"= caudanova$`Pr(>F)`[8],"gp_l.sd"= gpanova$`Pr(>F)`[7],"gp_r.sd"= gpanova$`Pr(>F)`[8],"put_l.sd"= putanova$`Pr(>F)`[7],"put_r.sd"= putanova$`Pr(>F)`[8])


P_CSHQ_total_INTERACTION <- P_CSHQ_total_INTERACTION %>%
 # Convert wide format to long format
 pivot_longer(
 cols = everything(),
 names_to = "term",
 values_to = "p_value"
 )

table_2_int_fx <- merge(df_interactions, P_CSHQ_total_INTERACTION, by = "term")
table_2_int_fx <- table_2_int_fx[order(table_2_int_fx$id), ]

new_int_df <- table_2_int_fx %>%
 # Create new columns
 mutate(
 # Remove .sd suffix and extract hemisphere
 hemisphere = ifelse(str_detect(term, "_l\\.sd"), "left", "right"),
 # Clean region names by removing hemisphere suffixes
 region = str_remove(term, "_[lr]\\.sd$")
 ) %>%
 # Reorder columns
 select(region, hemisphere, estimate, std.error, p_value) %>%
 mutate_at(vars(estimate, std.error), funs(round(., 2))) %>%
 mutate_at(vars(p_value), funs(round(., 3)))
