# Supplementary material for: Hypothalamic volume is associated with dysregulated sleep in autistic and non-autistic young children
Source: Autism. 2025 Jul 9;29(11):2885–97. doi: 10.1177/13623613251352249 (PMC12531386; doi:10.1177/13623613251352249)
Supplement: sj-docx-3-aut-10.1177_13623613251352249 – Supplemental material for Hypothalamic volume is associated with dysregulated sleep in autistic and non-autistic young children [file sj-docx-3-aut-10.1177_13623613251352249.docx]

Cleaned MI

Burt

2025-04-28

# Load required libraries
library(rio) # For data import/export

library(lavaan) # For structural equation modeling

library(semTools) # SEM tools

library(tidyverse) # For data manipulation and visualization

library(mice) # For multiple imputation

library(psych) # Various statistical functions

library(mitml) # Multiple imputation tools

library(miceadds) # Additional tools for 'mice'

library(car) # Companion to Applied Regression

library(readxl) # For reading Excel files

library(bestNormalize) # For data normalization

library(plyr) # For revaluing factors

library(finalfit)

library(naniar)

# -------------------------------
# Step 1: Load and process datasets
# -------------------------------

# Load raw data from CSV
df <- read.csv("MORI_raw.df_061920.csv")

# Load additional dataset and process it
dataset_2020_04_30 <- read_excel("dataset_2020-04-30.xlsx") %>%
 filter(visit == 1) %>% # Filter for first visit
 select(subj_id, demo_ch_rep_eth, demo_ch_rep_race, demo_ann_in) %>% # Select relevant columns
 mutate(
 # Create an ordered income category variable
 Income_ord = case_when(
 demo_ann_in %in% c("Under $10 000", "$10 000-$29 000", "$30 000-$49 000", "$50 000-$74 999", "$75 000-$99 999") ~ "Less than 100,000",
 demo_ann_in == "$100 000-$149 999" ~ "$100 000-$149 999",
 demo_ann_in == "$150 000 and above" ~ "$150 000 and above"
 ),
 Income_ord.n = as.ordered(case_when(
 demo_ann_in %in% c("Under $10 000", "$10 000-$29 000", "$30 000-$49 000", "$50 000-$74 999", "$75 000-$99 999") ~ 0,
 demo_ann_in == "$100 000-$149 999" ~ 1,
 demo_ann_in == "$150 000 and above" ~ 2
 )),
 # Handle missing race and ethnicity values
 demo_ch_rep_race = replace_na(demo_ch_rep_race, "Not reported"),
 demo_ch_rep_eth = replace_na(demo_ch_rep_eth, "Not reported"),
 # Group race into broader categories
 Race = case_when(
 demo_ch_rep_race == "African American/Black" ~ "African American/Black",
 demo_ch_rep_race == "Asian" ~ "Asian",
 demo_ch_rep_race %in% c("Mixed", "Other") ~ "Mixed/Other",
 demo_ch_rep_race %in% c("Not reported", "Refused") ~ "Refused/Not reported",
 demo_ch_rep_race == "White/Caucasian" ~ "White/Caucasian"
 )
 )

# Merge the processed dataset with the main data by subject ID
df <- dplyr::left_join(df, dataset_2020_04_30, by = "subj_id")

# -------------------------------
# Step 2: Recode and process sleep variables
# -------------------------------

# Revalue and order sleep variables
sleep_vars <- paste0("SLEEP_", c(1:12, 16:36, 40:48, 50, 52:54))
for (var in sleep_vars) {
 df[[var]] <- ordered(as.numeric(plyr::revalue(df[[var]], c(
 "Usually" = "3", "Sometimes" = "2", "Rarely" = "1",
 "N" = "1", "Very Sleepy" = "2", "Falls Asleep" = "3"
 ))))
}

# Reverse score selected sleep variables
reverse_scored_vars <- c("SLEEP_1", "SLEEP_2", "SLEEP_3", "SLEEP_8",
 "SLEEP_18", "SLEEP_19", "SLEEP_36", "SLEEP_40")
for (var in reverse_scored_vars) {
 df[[var]] <- ordered(4 - as.numeric(df[[var]]))
}

# -------------------------------
# Step 3: Specify variable formats
# -------------------------------

# Convert certain variables to factors or numeric
df$subj_id <- factor(df$subj_id)
df$gender <- factor(df$gender)
df$app_diagnosis <- factor(df$app_diagnosis)
df$ados_ccs <- as.numeric(df$ados_ccs)

# -------------------------------
# Step 4: Subset and merge additional data
# -------------------------------

# Select specific variables for analysis
df.sub.1 <- dplyr::select(df,
 subj_id, gender, app_diagnosis, scan_age, msel_dq,
 mori_total_volume, hippo_l, hippo_r, hypothalamus_l, hypothalamus_r,
 thalamus_l, thalamus_r, amyg_l, amyg_r, nucaccumbens_l, nucaccumbens_r,
 gp_l, gp_r, put_l, put_r, pons_l, pons_r, caud_l, caud_r,
 SLEEP_1, SLEEP_2, SLEEP_3, SLEEP_4, SLEEP_7, SLEEP_10,
 SLEEP_11, SLEEP_12, SLEEP_16, SLEEP_18, SLEEP_19, SLEEP_20,
 SLEEP_21, SLEEP_22, SLEEP_23, SLEEP_24, SLEEP_26, SLEEP_27, SLEEP_28, SLEEP_29,
 SLEEP_31, SLEEP_32, SLEEP_34, SLEEP_35,
 SLEEP_40, SLEEP_42, SLEEP_43, SLEEP_44, SLEEP_45, SLEEP_48,
 cbcl_emotionally_reactive_raw, cbcl_anxious_depressed_raw,
 cbcl_somatic_complaints_raw, cbcl_withdrawn_raw, cbcl_sleep_problem_raw,
 cbcl_attention_problem_raw, cbcl_aggressive_behavior_raw)

# Load SRS/SCQ dataset and normalize SCQ total scores
SRS_SCQ_df <- read_excel("SRS_SCQ_dataset_2023-04-20.xlsx")
SRS_SCQ_df$scq_tot_norm <- (orderNorm(SRS_SCQ_df$scq_total))$x.t

# Merge normalized SCQ scores into the main subset
df.sub.1 <- merge(df.sub.1, SRS_SCQ_df[, c("subj_id", "scq_tot_norm")], all.x = TRUE)

# Define explanatory and dependent variables

# Explanatory variables: These are the independent variables that will be used in the analysis.
explanatory <- c(
 "gender", "scan_age", "scq_tot_norm", "msel_dq", "mori_total_volume",
 "hippo_l", "hippo_r", "hypothalamus_l", "hypothalamus_r",
 "thalamus_l", "thalamus_r", "amyg_l", "amyg_r", "nucaccumbens_l", "nucaccumbens_r",
 "gp_l", "gp_r", "put_l", "put_r", "pons_l", "pons_r", "caud_l", "caud_r",
 "SLEEP_1", "SLEEP_2", "SLEEP_16", "SLEEP_19", "SLEEP_22", "SLEEP_35", "SLEEP_48"
)

# Dependent variables: These are the outcome variables that will be examined.
dependent <- c(
 "cbcl_aggressive_behavior_raw", "cbcl_attention_problem_raw", "cbcl_emotionally_reactive_raw",
 "cbcl_anxious_depressed_raw", "cbcl_withdrawn_raw", "cbcl_somatic_complaints_raw"
)

# Analyze the missing data pattern for dependent and explanatory variables
# The `missing_pattern` function (assumed to be from a custom or specific library)
# identifies patterns of missing data in the provided variables.
df.sub.1 %>%
 missing_pattern(dependent, explanatory)

pct_miss_case(df.sub.1[,c("cbcl_aggressive_behavior_raw", "cbcl_attention_problem_raw", "cbcl_emotionally_reactive_raw",
 "cbcl_anxious_depressed_raw",
 "cbcl_withdrawn_raw",
 "cbcl_somatic_complaints_raw")])

## [1] 9.491525

miss_case_table(df.sub.1[,c("cbcl_aggressive_behavior_raw", "cbcl_attention_problem_raw", "cbcl_emotionally_reactive_raw",
 "cbcl_anxious_depressed_raw",
 "cbcl_withdrawn_raw",
 "cbcl_somatic_complaints_raw")])

dependent = "cbcl_aggressive_behavior_raw"
Agg_mis_tap <- df.sub.1 %>%
 missing_compare(dependent, explanatory)

dependent = "cbcl_attention_problem_raw"
Att_mis_tap <- df.sub.1 %>%
 missing_compare(dependent, explanatory)

dependent = "cbcl_emotionally_reactive_raw"
ER_mis_tap <- df.sub.1 %>%
 missing_compare(dependent, explanatory)

dependent = "cbcl_anxious_depressed_raw"
Anx_Dep_mis_tap <- df.sub.1 %>%
 missing_compare(dependent, explanatory)

dependent = "cbcl_withdrawn_raw"
Withdraw_mis_tap <- df.sub.1 %>%
 missing_compare(dependent, explanatory)

dependent = "cbcl_somatic_complaints_raw"
Somat_mis_tap <- df.sub.1 %>%
 missing_compare(dependent, explanatory)

Missing data for the ASD group

df.sub.ASD <- df.sub.1 %>% filter(app_diagnosis == "ASD")

dependent = "cbcl_aggressive_behavior_raw"
Agg_mis_tap <- df.sub.ASD %>%
 missing_compare(dependent, explanatory)

dependent = "cbcl_attention_problem_raw"
Att_mis_tap <- df.sub.ASD %>%
 missing_compare(dependent, explanatory)

dependent = "cbcl_emotionally_reactive_raw"
ER_mis_tap <- df.sub.ASD %>%
 missing_compare(dependent, explanatory)

dependent = "cbcl_anxious_depressed_raw"
Anx_Dep_mis_tap <- df.sub.ASD %>%
 missing_compare(dependent, explanatory)

dependent = "cbcl_withdrawn_raw"
Withdraw_mis_tap <- df.sub.ASD %>%
 missing_compare(dependent, explanatory)

dependent = "cbcl_somatic_complaints_raw"
Somat_mis_tap <- df.sub.ASD %>%
 missing_compare(dependent, explanatory)

# Imputation Script for ASD and TD Groups Using MICE

# -------------------------------
# Step 1: Imputation for ASD Group
# -------------------------------

# Subset the data for ASD group
df.sub.ASD <- df.sub.1[df.sub.1$app_diagnosis == "ASD", ]

# Initialize MICE with no iterations to extract methods and predictors
ini.ASD <- mice(df.sub.ASD, maxit = 0, print = FALSE)

## Warning: Number of logged events: 1

# Extract initial predictor matrix and imputation methods
pred.ASD <- ini.ASD$pred
meth.ASD <- ini.ASD$meth

# Update imputation methods for ordinal variables
# Use CART (Classification and Regression Trees) as it is robust for ordinal data
meth.ASD <- plyr::mapvalues(meth.ASD, "polr", "cart") # Replace "polr" with "cart" for ordinal variables
meth.ASD["msel_dq"] <- "cart"
meth.ASD["cbcl_emotionally_reactive_raw"] <- "cart"
meth.ASD["cbcl_anxious_depressed_raw"] <- "cart"
meth.ASD["cbcl_somatic_complaints_raw"] <- "cart"
meth.ASD["cbcl_withdrawn_raw"] <- "cart"
meth.ASD["cbcl_sleep_problem_raw"] <- "cart"
meth.ASD["cbcl_attention_problem_raw"] <- "cart"
meth.ASD["cbcl_aggressive_behavior_raw"] <- "cart"
meth.ASD["scq_tot_norm"] <- "cart"

# Remove unnecessary predictors
# Exclude `subj_id` (ID variable) and `app_diagnosis` (grouping variable) from the predictor matrix
pred.ASD[, "subj_id"] <- 0
pred.ASD[, "app_diagnosis"] <- 0

# Perform multiple imputations
imp.ASD <- mice(
 df.sub.ASD,
 m = 200, # Number of imputations
 maxit = 40, # Maximum number of iterations
 meth = meth.ASD, # Updated imputation methods
 pred = pred.ASD, # Updated predictor matrix
 printFlag = FALSE
)

# Summarize and visualize the imputation results for ASD group
summary(imp.ASD)

plot(imp.ASD)

# -------------------------------
# Step 2: Imputation for TD Group
# -------------------------------

# Subset the data for TD group
df.sub.TD <- df.sub.1[df.sub.1$app_diagnosis == "TD", ]

# Initialize MICE with no iterations to extract methods and predictors
ini.TD <- mice(df.sub.TD, maxit = 0, print = FALSE)

## Warning: Number of logged events: 1

# Extract initial predictor matrix and imputation methods
pred.TD <- ini.TD$pred
meth.TD <- ini.TD$meth

# Update imputation methods for ordinal variables
meth.TD <- plyr::mapvalues(meth.TD, "polr", "cart") # Replace "polr" with "cart" for ordinal variables
meth.TD["msel_dq"] <- "cart"
meth.TD["cbcl_emotionally_reactive_raw"] <- "cart"
meth.TD["cbcl_anxious_depressed_raw"] <- "cart"
meth.TD["cbcl_somatic_complaints_raw"] <- "cart"
meth.TD["cbcl_withdrawn_raw"] <- "cart"
meth.TD["cbcl_sleep_problem_raw"] <- "cart"
meth.TD["cbcl_attention_problem_raw"] <- "cart"
meth.TD["cbcl_aggressive_behavior_raw"] <- "cart"
meth.TD["scq_tot_norm"] <- "cart"

# Remove unnecessary predictors
# Exclude `subj_id` (ID variable) and `app_diagnosis` (grouping variable) from the predictor matrix
pred.TD[, "subj_id"] <- 0
pred.TD[, "app_diagnosis"] <- 0

# Perform multiple imputations
imp.TD <- mice(
 df.sub.TD,
 m = 200, # Number of imputations
 maxit = 40, # Maximum number of iterations
 meth = meth.TD, # Updated imputation methods
 pred = pred.TD, # Updated predictor matrix
 printFlag = TRUE
)

# -------------------------------
# Step 3: Combine Imputed Datasets
# -------------------------------

# Combine imputed datasets from ASD and TD groups into a single mids object
imp <- rbind(imp.ASD, imp.TD)

# Save the combined imputed dataset for future use
save(imp, file = "imp.RData")

# Process and Analyze Imputed Data

# -------------------------------
# Step 1: Load the imputed dataset
# -------------------------------
# Load the saved imputed dataset
load("imp.RData")

# Convert the imputed dataset to a long format
long1 <- complete(imp, action = 'long', include = TRUE)

# -------------------------------
# Step 2: Convert sleep variables to numeric
# -------------------------------
# Convert sleep variables to numeric for further analysis
sleep_vars <- c(
 "SLEEP_1", "SLEEP_2", "SLEEP_3", "SLEEP_4", "SLEEP_7", "SLEEP_10",
 "SLEEP_11", "SLEEP_12", "SLEEP_16", "SLEEP_18", "SLEEP_19", "SLEEP_20",
 "SLEEP_21", "SLEEP_22", "SLEEP_23", "SLEEP_24", "SLEEP_26", "SLEEP_27",
 "SLEEP_28", "SLEEP_29", "SLEEP_31", "SLEEP_32", "SLEEP_34", "SLEEP_35",
 "SLEEP_40", "SLEEP_42", "SLEEP_43", "SLEEP_44", "SLEEP_45", "SLEEP_48"
)

long1[sleep_vars] <- lapply(long1[sleep_vars], as.numeric)

# Convert SCQ normalized total to numeric
long1$scq_total <- as.numeric(long1$scq_tot_norm)

# -------------------------------
# Step 3: Create derived variables
# -------------------------------
# Compute aggregate scores for externalizing, internalizing, and sleep behaviors
long1 <- long1 %>%
 dplyr::mutate(
 Externalizing_Raw = rowSums(long1[, c("cbcl_aggressive_behavior_raw", "cbcl_attention_problem_raw")], na.rm = TRUE),
 Internalizing_Raw = rowSums(long1[, c(
 "cbcl_emotionally_reactive_raw", "cbcl_anxious_depressed_raw",
 "cbcl_withdrawn_raw", "cbcl_somatic_complaints_raw"
 )], na.rm = TRUE),
 Sleep_sum = rowSums(long1[, c("SLEEP_1", "SLEEP_2", "SLEEP_16", "SLEEP_19", "SLEEP_22", "SLEEP_35", "SLEEP_48")], na.rm = TRUE)
 )

# -------------------------------
# Step 4: Standardize and transform variables
# -------------------------------
# Convert categorical variables to ordered factors
long1$gender.ord <- ordered(long1$gender)
long1$diagnosis.ord <- ordered(long1$app_diagnosis)

# Standardize and transform continuous variables
long1$log_cshq_sum_sd <- as.vector(scale(log(long1$Sleep_sum)))
long1$hypothalamus_l.sd <- as.vector(scale(long1$hypothalamus_l))
long1$hypothalamus_r.sd <- as.vector(scale(long1$hypothalamus_r))
long1$Externalizing_Raw.sd <- as.vector(scale(long1$Externalizing_Raw))
long1$Internalizing_Raw.sd <- as.vector(scale(long1$Internalizing_Raw))
long1$mori_total_volume.sd <- as.vector(scale(long1$mori_total_volume))
long1$scq_tot_norm_sd <- as.vector(scale(long1$scq_tot_norm))


# Convert the entire dataset back to mids object
imp2 <- as.mids(long1)

# -------------------------------
# Step 6: Visualize imputations
# -------------------------------
# Generate density plots to check the distribution of imputed variables
#densityplot(imp2, ~cbcl_emotionally_reactive_raw)
#densityplot(imp2, ~cbcl_anxious_depressed_raw)
#densityplot(imp2, ~cbcl_somatic_complaints_raw)
#densityplot(imp2, ~cbcl_withdrawn_raw)
#densityplot(imp2, ~cbcl_sleep_problem_raw)
#densityplot(imp2, ~cbcl_aggressive_behavior_raw)
#densityplot(imp2, ~cbcl_attention_problem_raw)
#densityplot(imp2, ~Externalizing_Raw.sd)
#densityplot(imp2, ~Internalizing_Raw.sd)
#densityplot(imp2, ~scq_tot_norm)

# -------------------------------
# Step 7: Save the processed imputed dataset
# -------------------------------
# Save the processed imputed dataset for future use
save(imp2, file = "imp2.RData")
