# Supplementary material for: Hypothalamic volume is associated with dysregulated sleep in autistic and non-autistic young children
Source: Autism. 2025 Jul 9;29(11):2885–97. doi: 10.1177/13623613251352249 (PMC12531386; doi:10.1177/13623613251352249)
Supplement: sj-docx-4-aut-10.1177_13623613251352249 – Supplemental material for Hypothalamic volume is associated with dysregulated sleep in autistic and non-autistic young children [file sj-docx-4-aut-10.1177_13623613251352249.docx]

Cleaned Mediation

Burt

2025-04-29

Summary statistics for the following analyses are provided in Table 3, Table S3

# R Script for Multiple Imputation, ANOVA, and Regression Analysis

# -------------------------------
# Load Required Libraries
# -------------------------------
library(rio) # Import/export data

library(devtools) # For installing packages from GitHub

library(lavaan) # Structural Equation Modeling

library(semTools) # SEM utility functions

library(tidyverse) # Data manipulation and visualization

library(mice) # Multiple imputation

library(psych) # Statistical functions

library(mitml) # Multiple imputation tools

library(miceadds) # Additional tools for 'mice'

library(huxtable) # Pretty tables

library(data.table) # Fast data manipulation

library(car) # Companion to Applied Regression

library(flextable)

# -------------------------------
# Step 1: Load Imputed Data
# -------------------------------
df.mi <- import("imp2.RData") # Load multiple imputed datasets

## Warning: Missing `trust` will be set to FALSE by default for RData in 2.0.0.

# Convert mids object to long format for easier manipulation
long2 <- complete(df.mi, action = 'long', include = TRUE)

# -------------------------------
# Step 2: Recode Variables
# -------------------------------
# Re-level app_diagnosis to make "TD" the reference group
long2$app_diagnosis <- fct_relevel(long2$app_diagnosis, "TD", "ASD")

# Convert to a list format for miceadds functions

df.mi2 <- as.mids(long2)

imp.list2 <- miceadds::datlist_create(df.mi2)

Presented in Table 3

# Define the mediation model as a string
# This model includes equations for Externalizing_Raw.sd and log_cshq_sum_sd,
# with hypothesized relationships and an indirect mediation effect.
Ex.med <- '

# Equation for Externalizing_Raw.sd
# Models Externalizing_Raw.sd as a function of app_diagnosis, hypothalamus volumes (left and right),
# gender, scan age, and total brain volume (mori_total_volume.sd).
Externalizing_Raw.sd ~ 1 + app_diagnosis + hypothalamus_l.sd + a2*hypothalamus_r.sd + gender + scan_age + mori_total_volume.sd

# Equation for log_cshq_sum_sd
# Models log_cshq_sum_sd as a function of Externalizing_Raw.sd, app_diagnosis, hypothalamus volumes,
# gender, scan age, and total brain volume.
log_cshq_sum_sd ~ 1 + b1*Externalizing_Raw.sd + app_diagnosis + hypothalamus_l.sd + c1*hypothalamus_r.sd + gender + scan_age + mori_total_volume.sd

# Define the mediation effect (indirect effect)
# HypR.Ext.Med represents the indirect effect of hypothalamus_r.sd on log_cshq_sum_sd via Externalizing_Raw.sd.
HypR.Ext.Med := a2 * b1

'

# Fit the mediation model using multiple imputed datasets
# The lavaan.mi package is used for SEM with multiple imputations.
Ex.med.fit <- lavaan.mi::sem.mi(
 model = Ex.med, # Specify the SEM model as a string
 estimator = "MLR", # Use Maximum Likelihood with Robust standard errors (MLR)
 fixed.x = TRUE, # Treat observed exogenous variables as fixed
 # orthoganal.x = FALSE, # Uncomment if orthogonal exogenous variables are needed
 data = imp.list2 # Provide the list of imputed datasets
)

# Generate a summary of the fitted SEM model
summary(Ex.med.fit)

# Extract the model coefficients
Ex.med.coef <- coef(Ex.med.fit) # Coefficients of the model

# Extract the variance-covariance matrix of the model coefficients
Ex.med.vcov <- vcov(Ex.med.fit) # Variance-covariance matrix of the coefficients

# Perform Monte Carlo confidence interval estimation for specific parameters
monteCarloCI(
 expr = c(a = 'a2', # Define the parameter 'a2'
 b = 'b1', # Define the parameter 'b1'
 c = 'c1', # Define the parameter 'c1'
 ind = 'a2*b1'), # Define the indirect mediation effect as 'a2*b1'
 coefs = Ex.med.coef, # Provide the coefficients from the fitted model
 ACM = Ex.med.vcov, # Provide the variance-covariance matrix
 plot = TRUE, # Generate a plot of the confidence intervals
 ask = TRUE # Pause and prompt the user before proceeding
)

Presented in Table S3

# Define the mediation model as a string
# This model includes group-specific parameters to analyze differences between groups.
Ex.med <- '

# Model for Externalizing_Raw.sd
# Models Externalizing_Raw.sd as a function of left/right hypothalamus volumes (hypothalamus_l.sd, hypothalamus_r.sd),
# gender, scan age, and total brain volume (mori_total_volume.sd), with group-specific coefficients for hypothalamus_r.sd (AA_hr, AB_hr).
Externalizing_Raw.sd ~ 1 + hypothalamus_l.sd + c(AA_hr, AB_hr)*hypothalamus_r.sd + gender + scan_age + mori_total_volume.sd

# Model for log_cshq_sum_sd
# Models log_cshq_sum_sd as a function of Externalizing_Raw.sd (group-specific coefficients BA_Ex and BB_Ex),
# hypothalamus volumes, gender, scan age, and total brain volume, with additional group-specific coefficients for hypothalamus_r.sd (BA_hr, BB_hr).
log_cshq_sum_sd ~ 1 + c(BA_Ex, BB_Ex)*Externalizing_Raw.sd + hypothalamus_l.sd + c(BA_hr, BB_hr)*hypothalamus_r.sd + gender + scan_age + mori_total_volume.sd

# Define indirect effects for each group
# IndirectRH.A and IndirectRH.B represent the indirect effects of hypothalamus_r.sd on log_cshq_sum_sd
# via Externalizing_Raw.sd for groups A and B, respectively.
IndirectRH.A := AA_hr * BA_Ex
IndirectRH.B := AB_hr * BB_Ex

'

# Fit the mediation model using multiple imputed datasets
# sem.mi is used for SEM analysis when multiple imputations are involved.
out2 <- sem.mi(
 model = Ex.med, # Specify the SEM model as a string
 estimator = "MLR", # Use Maximum Likelihood with Robust standard errors (MLR)
 fixed.x = TRUE, # Treat observed exogenous variables as fixed
 # orthoganal.x = FALSE, # Uncomment if orthogonal exogenous variables are needed
 data = imp.list2, # Provide the list of imputed datasets
 group = "app_diagnosis" # Perform group-specific analysis based on "app_diagnosis"
)

# Perform Wald tests to compare constrained parameters across groups
# Test if the group-specific coefficients for hypothalamus_r.sd in Externalizing_Raw.sd are equal
lavTestWald.mi(out2, constraints = "AA_hr == AB_hr")

# Test if the group-specific coefficients for Externalizing_Raw.sd in log_cshq_sum_sd are equal
lavTestWald.mi(out2, constraints = "BA_Ex == BB_Ex")

# Test if the group-specific coefficients for hypothalamus_r.sd in log_cshq_sum_sd are equal
lavTestWald.mi(out2, constraints = "BA_hr == BB_hr")

# Test if the indirect effects (IndirectRH.A and IndirectRH.B) are equal across groups
lavTestWald.mi(out2, constraints = "IndirectRH.A == IndirectRH.B")

### ——————————-

Analyses for internalizing symptoms

Int.med <- '

Internalizing_Raw.sd ~ 1 + app_diagnosis + a2*hypothalamus_r.sd + hypothalamus_l.sd + gender + scan_age + mori_total_volume.sd

'

Int.med.fit <- lavaan.mi::sem.mi(Int.med,
 estimator = "MLR",
 fixed.x = TRUE,
 #orthoganal.x = FALSE,
 data = imp.list2
 )

summary(Int.med.fit)
