# Supplementary material for: Hypothalamic volume is associated with dysregulated sleep in autistic and non-autistic young children
Source: Autism. 2025 Jul 9;29(11):2885–97. doi: 10.1177/13623613251352249 (PMC12531386; doi:10.1177/13623613251352249)
Supplement: sj-docx-5-aut-10.1177_13623613251352249 – Supplemental material for Hypothalamic volume is associated with dysregulated sleep in autistic and non-autistic young children [file sj-docx-5-aut-10.1177_13623613251352249.docx]

Cleaned code

Burt

2025-04-28

This document contains the cleaned code for the analysis of the MORI dataset. The analysis includes data import, preprocessing, descriptive statistics, regression modeling, and hypothesis testing.

Analyses and output are for Table 1 and Table 2 in the manuscript and Table S1 and Table S2 in the supplementary methods section.

# Load required libraries
library(rio) # For data import/export

library(lavaan) # For structural equation modeling

library(semTools) # SEM tools

library(tidyverse) # For data manipulation and visualization

library(mice) # For multiple imputation

library(psych) # Various statistical functions

library(mitml) # Multiple imputation tools

library(miceadds) # Additional tools for 'mice'

library(car) # Companion to Applied Regression

library(readxl) # For reading Excel files

# Load raw data from CSV
df <- read.csv("MORI_raw.df_061920.csv")

# Load additional dataset and process it
dataset_2020_04_30 <- read_excel("dataset_2020-04-30.xlsx") %>%
 filter(visit == 1) %>% # Filter for first visit
 select(subj_id, demo_ch_rep_eth, demo_ch_rep_race, demo_ann_in) %>% # Select relevant columns
 mutate(
 # Create an ordered income category variable
 Income_ord = case_when(
 demo_ann_in %in% c("Under $10 000", "$10 000-$29 000", "$30 000-$49 000", "$50 000-$74 999", "$75 000-$99 999") ~ "Less than 100,000",
 demo_ann_in == "$100 000-$149 999" ~ "$100 000-$149 999",
 demo_ann_in == "$150 000 and above" ~ "$150 000 and above"
 ),
 Income_ord.n = as.ordered(case_when(
 demo_ann_in %in% c("Under $10 000", "$10 000-$29 000", "$30 000-$49 000", "$50 000-$74 999", "$75 000-$99 999") ~ 0,
 demo_ann_in == "$100 000-$149 999" ~ 1,
 demo_ann_in == "$150 000 and above" ~ 2
 )),
 # Handle missing race and ethnicity values
 demo_ch_rep_race = replace_na(demo_ch_rep_race, "Not reported"),
 demo_ch_rep_eth = replace_na(demo_ch_rep_eth, "Not reported"),
 # Group race into broader categories
 Race = case_when(
 demo_ch_rep_race == "African American/Black" ~ "African American/Black",
 demo_ch_rep_race == "Asian" ~ "Asian",
 demo_ch_rep_race %in% c("Mixed", "Other") ~ "Mixed/Other",
 demo_ch_rep_race %in% c("Not reported", "Refused") ~ "Refused/Not reported",
 demo_ch_rep_race == "White/Caucasian" ~ "White/Caucasian"
 )
 )

# Merge the processed dataset with the main data by subject ID
df <- dplyr::left_join(df, dataset_2020_04_30, by = "subj_id")

# Display column names of the processed dataset
names(dataset_2020_04_30)

# Specify variable formats
df$subj_id <- factor(df$subj_id)
df$gender <- factor(df$gender)
df$app_diagnosis <- factor(df$app_diagnosis)
df$ados_ccs <- as.numeric(df$ados_ccs)

# Select a subset of variables for analysis
df.sub.1 <- dplyr::select(
 df,
 subj_id, demo_ch_rep_eth, demo_ch_rep_race, demo_ann_in, Income_ord, Income_ord.n, Race,
 gender, app_diagnosis, scan_age, msel_dq,
 mori_total_volume, hippo_l, hippo_r, hypothalamus_l, hypothalamus_r,
 thalamus_l, thalamus_r, amyg_l, amyg_r, nucaccumbens_l, nucaccumbens_r,
 gp_l, gp_r, put_l, put_r, pons_l, pons_r, caud_l, caud_r,
 SLEEP_1:SLEEP_48, cshq_sum, cshq_total,
 cbcl_emotionally_reactive_raw, cbcl_anxious_depressed_raw,
 cbcl_somatic_complaints_raw, cbcl_withdrawn_raw, cbcl_sleep_problem_raw,
 cbcl_attention_problem_raw, cbcl_aggressive_behavior_raw, cbcl_externalizing_raw,
 cbcl_internalizing_raw, cbcl_externalizing_t, cbcl_internalizing_t, ados_ccs
)

# Generate descriptive statistics (Table 1)
# Summarize data by app_diagnosis and include selected variables
library(gtsummary)
df.sub.1 |>
 tbl_summary(
 by = app_diagnosis,
 include = c(gender, scan_age, demo_ch_rep_eth, Race, Income_ord, msel_dq, ados_ccs, cshq_sum, cshq_total, cbcl_externalizing_t, cbcl_internalizing_t),
 statistic = list(
 all_continuous() ~ "{mean} ({sd})", # Mean and standard deviation for continuous variables
 all_categorical() ~ "{n} / {N} ({p}%)" # Proportion for categorical variables
 ),
 digits = all_continuous() ~ 2,
 missing_text = "(Missing)" # Label for missing data
 )

### Main analyses

# Standardize and transform variables for regression models
df.sub.1$cshq_sum.sd <- scale(log(df.sub.1$cshq_sum), center = TRUE, scale = TRUE)
df.sub.1 <- df.sub.1 %>%
 mutate(across(
 c(hypothalamus_l, hypothalamus_r, hippo_l, hippo_r, thalamus_l, thalamus_r,
 amyg_l, amyg_r, nucaccumbens_l, nucaccumbens_r, gp_l, gp_r, put_l, put_r,
 pons_l, pons_r, caud_l, caud_r),
 ~ scale(.x, center = TRUE, scale = TRUE),
 .names = "{col}.sd"
 ))

# Create binary variable for diagnosis (1 = ASD, 0 = Other)
#df.sub.1$app_diagnosis.YN <- ifelse(df.sub.1$app_diagnosis == "ASD", 1, 0)

# Run multiple linear regression models for each brain region
library(broom)

lmhypothallog <- lm(cshq_sum.sd ~ gender + scan_age + mori_total_volume + app_diagnosis * (hypothalamus_l.sd + hypothalamus_r.sd),df.sub.1)
hypothalanova <- Anova(lmhypothallog,type = "II")


lmhippolog <- lm(cshq_sum.sd ~ gender + scan_age + mori_total_volume + app_diagnosis * (hippo_l.sd + hippo_r.sd),df.sub.1)
hippoanova <- Anova(lmhippolog,type = "II")

lmthalamuslog <- lm(cshq_sum.sd ~ gender + scan_age + mori_total_volume + app_diagnosis * (thalamus_l.sd + thalamus_r.sd),df.sub.1)
thalamusanova <- Anova(lmthalamuslog,type = "II")

lmamyglog <- lm(cshq_sum.sd ~ gender + scan_age + mori_total_volume + app_diagnosis * (amyg_l.sd + amyg_r.sd),df.sub.1)
amyganova <- Anova(lmamyglog,type = "II")

lmnucaccumbenslog <- lm(cshq_sum.sd ~ gender + scan_age + mori_total_volume + app_diagnosis * (nucaccumbens_l.sd + nucaccumbens_r.sd),df.sub.1)
nucaccumbensanova <- Anova(lmnucaccumbenslog,type = "II")

lmponslog <- lm(cshq_sum.sd ~ gender + scan_age + mori_total_volume + app_diagnosis * (pons_l.sd + pons_r.sd),df.sub.1)
ponsanova <- Anova(lmponslog,type = "II")

lmcaudlog <- lm(cshq_sum.sd ~ gender + scan_age + mori_total_volume + app_diagnosis * (caud_l.sd + caud_r.sd),df.sub.1)
caudanova <- Anova(lmcaudlog,type = "II")

lmgplog <- lm(cshq_sum.sd ~ gender + scan_age + mori_total_volume + app_diagnosis * (gp_l.sd + gp_r.sd),df.sub.1)
gpanova <- Anova(lmgplog,type = "II")

lmputlog <- lm(cshq_sum.sd ~ gender + scan_age + mori_total_volume + app_diagnosis * (put_l.sd + put_r.sd),df.sub.1)
putanova <- Anova(lmputlog,type = "II")

# Create a list of regression models for different brain regions
# Each model corresponds to a brain region and is stored with a descriptive name
models <- list(
 hypothalamus = lmhypothallog, # Model for hypothalamus
 hippocampus = lmhippolog, # Model for hippocampus
 thalamus = lmthalamuslog, # Model for thalamus
 amygdala = lmamyglog, # Model for amygdala
 nucaccumbens = lmnucaccumbenslog, # Model for nucleus accumbens
 pons = lmponslog, # Model for pons
 caudate = lmcaudlog, # Model for caudate
 gp = lmgplog, # Model for globus pallidus
 putamen = lmputlog # Model for putamen
)

# Extract regression coefficients (betas) and standard errors for main effects
# This focuses on the main effects of the brain regions (assumed to be 6th and 7th terms in each model)
main_results <- lapply(names(models), function(region_name) {
 tidy_mod <- broom::tidy(models[[region_name]]) %>%
 select(term, estimate, std.error) # Keep only the term, estimate, and std.error columns
 tidy_mod[c(6, 7), ] # Extract rows corresponding to main effects of the brain region
})

# Combine the results into a single data frame
df <- as.data.frame(do.call(rbind, main_results))

# Add an ID column to the data frame to maintain the order of terms
df$id <- 1:nrow(df)

# Extract p-values for main effects from the ANOVA results
# Note: Ensure the ANOVA objects (e.g., hypothalanova, hippoanova) are available in your environment
P_CSHQ_SUM <- data.frame(
 "hypothalamus_l.sd" = hypothalanova$`Pr(>F)`[5],
 "hypothalamus_r.sd" = hypothalanova$`Pr(>F)`[6],
 "hippo_l.sd" = hippoanova$`Pr(>F)`[5],
 "hippo_r.sd" = hippoanova$`Pr(>F)`[6],
 "thalamus_l.sd" = thalamusanova$`Pr(>F)`[5],
 "thalamus_r.sd" = thalamusanova$`Pr(>F)`[6],
 "amyg_l.sd" = amyganova$`Pr(>F)`[5],
 "amyg_r.sd" = amyganova$`Pr(>F)`[6],
 "nucaccumbens_l.sd" = nucaccumbensanova$`Pr(>F)`[5],
 "nucaccumbens_r.sd" = nucaccumbensanova$`Pr(>F)`[6],
 "pons_l.sd" = ponsanova$`Pr(>F)`[5],
 "pons_r.sd" = ponsanova$`Pr(>F)`[6],
 "caud_l.sd" = caudanova$`Pr(>F)`[5],
 "caud_r.sd" = caudanova$`Pr(>F)`[6],
 "gp_l.sd" = gpanova$`Pr(>F)`[5],
 "gp_r.sd" = gpanova$`Pr(>F)`[6],
 "put_l.sd" = putanova$`Pr(>F)`[5],
 "put_r.sd" = putanova$`Pr(>F)`[6]
)

# Adjust p-values for multiple comparisons using the Benjamini-Hochberg procedure
BHP_CSHQ_SUM <- p.adjust(as.vector(P_CSHQ_SUM), method = "BH")

# Create a data frame with the adjusted p-values and their corresponding terms
BH_p_corrected_CSHQ_SUM <- data.frame(
 "BH_p_corrected_CSHQ_SUM" = BHP_CSHQ_SUM
)
BH_p_corrected_CSHQ_SUM$term <- rownames(BH_p_corrected_CSHQ_SUM)

# Merge the regression results (betas and standard errors) with the adjusted p-values
table_2_main_fx <- merge(df, BH_p_corrected_CSHQ_SUM, by = "term")

# Reorder the merged data frame based on the ID column
table_2_main_fx <- table_2_main_fx[order(table_2_main_fx$id), ]

# Clean and reorganize the data for presentation in a table
new_df <- data.frame(table_2_main_fx[, -4], row.names = table_2_main_fx[, 4]) %>%
 rename(p_value = BH_p_corrected_CSHQ_SUM) %>% # Rename the adjusted p-value column
 mutate(
 # Extract the brain region name by removing the hemisphere suffix (_l.sd or _r.sd)
 region = str_remove(term, "_[lr]\\.sd$"),
 # Define the hemisphere based on the suffix in the term
 hemisphere = ifelse(str_detect(term, "_l\\.sd$"), "left", "right")
 ) %>%
 select(region, hemisphere, estimate, std.error, p_value) %>% # Select relevant columns
 mutate_at(vars(estimate, std.error), funs(round(., 2))) %>% # Round estimates and standard errors to 2 decimal places
 mutate_at(vars(p_value), funs(round(., 3))) # Round p-values to 3 decimal places

# Create summary table for interaction terms

main_results_int <- lapply(names(models), function(region_name) {
 tidy_mod <- broom::tidy(models[[region_name]]) %>%
 select(term, estimate, std.error)
 tidy_mod[c(8,9),]
})

df_interactions <- as.data.frame(do.call(rbind, main_results_int))


df_interactions$term <- str_remove(df_interactions$term , "app_diagnosisTD:")

df_interactions$id <- 1:nrow(df_interactions)


P_CSHQ_SUM_INTERACTION <-data.frame("hypothalamus_l.sd"=hypothalanova$`Pr(>F)`[7],"hypothalamus_r.sd"=hypothalanova$`Pr(>F)`[8],"hippo_l.sd"=hippoanova$`Pr(>F)`[7],"hippo_r.sd"= hippoanova$`Pr(>F)`[8],"thalamus_l.sd"= thalamusanova$`Pr(>F)`[7],"thalamus_r.sd"= thalamusanova$`Pr(>F)`[8],"amyg_l.sd"= amyganova$`Pr(>F)`[7],"amyg_r.sd"= amyganova$`Pr(>F)`[8],"nucaccumbens_l.sd"= nucaccumbensanova$`Pr(>F)`[7],"nucaccumbens_r.sd"= nucaccumbensanova$`Pr(>F)`[8],"pons_l.sd"= ponsanova$`Pr(>F)`[7],"pons_r.sd"= ponsanova$`Pr(>F)`[8],"caud_l.sd"= caudanova$`Pr(>F)`[7],"caud_r.sd"= caudanova$`Pr(>F)`[8],"gp_l.sd"= gpanova$`Pr(>F)`[7],"gp_r.sd"= gpanova$`Pr(>F)`[8],"put_l.sd"= putanova$`Pr(>F)`[7],"put_r.sd"= putanova$`Pr(>F)`[8])


P_CSHQ_SUM_INTERACTION <- P_CSHQ_SUM_INTERACTION %>%
 # Convert wide format to long format
 pivot_longer(
 cols = everything(),
 names_to = "term",
 values_to = "p_value"
 )

table_2_int_fx <- merge(df, P_CSHQ_SUM_INTERACTION, by = "term")
table_2_int_fx <- table_2_int_fx[order(table_2_int_fx$id), ]

new_int_df <- table_2_int_fx %>%
 # Create new columns
 mutate(
 # Remove .sd suffix and extract hemisphere
 hemisphere = ifelse(str_detect(term, "_l\\.sd"), "left", "right"),
 # Clean region names by removing hemisphere suffixes
 region = str_remove(term, "_[lr]\\.sd$")
 ) %>%
 # Reorder columns
 select(region, hemisphere, estimate, std.error, p_value) %>%
 mutate_at(vars(estimate, std.error), funs(round(., 2))) %>%
 mutate_at(vars(p_value), funs(round(., 3)))

### Check ROI by sex and ROI by sex by diagnosis interactions

# LRT ROI by SEX by DIAGNOSIS

#Hypothalamus * Sex * Diagnosis
lmhyposexdiag <- lm(cshq_sum.sd ~ scan_age + mori_total_volume + gender + gender * app_diagnosis * (hypothalamus_l.sd + hypothalamus_r.sd),df.sub.1)
lmhyposexdiag_null <- lm(cshq_sum.sd ~ scan_age + mori_total_volume + gender * app_diagnosis + gender * (hypothalamus_l.sd + hypothalamus_r.sd) + app_diagnosis * (hypothalamus_l.sd + hypothalamus_r.sd),df.sub.1)
LRT_hyposexdiag <- anova(lmhyposexdiag,lmhyposexdiag_null)

#Hippocampus * Sex * Diagnosis
lmhipposexdiag <- lm(cshq_sum.sd ~ scan_age + mori_total_volume + gender * app_diagnosis * ( hippo_l.sd + hippo_r.sd),df.sub.1)
lmhipposexdiag_null <- lm(cshq_sum.sd ~ scan_age + mori_total_volume + gender * app_diagnosis + gender * (hippo_l.sd + hippo_r.sd) + app_diagnosis * (hippo_l.sd + hippo_r.sd),df.sub.1)
LRT_hipposexdiag <- anova(lmhipposexdiag,lmhipposexdiag_null)

#Thalamus * Sex * Diagnosis
lmthalamussexdiag <- lm(cshq_sum.sd ~ scan_age + mori_total_volume + gender * app_diagnosis * ( thalamus_l.sd + thalamus_r.sd),df.sub.1)
lmthalamussexdiag_null <- lm(cshq_sum.sd ~ scan_age + mori_total_volume + gender * app_diagnosis + gender * (thalamus_l.sd + thalamus_r.sd) + app_diagnosis * (thalamus_l.sd + thalamus_r.sd),df.sub.1)
LRT_thalamussexdiag <- anova(lmthalamussexdiag,lmthalamussexdiag_null)

#Amygdala * Sex * Diagnosis
lmamygsexdiag <- lm(cshq_sum.sd ~ scan_age + mori_total_volume + gender * app_diagnosis * ( amyg_l.sd + amyg_r.sd),df.sub.1)
lmamygsexdiag_null <- lm(cshq_sum.sd ~ scan_age + mori_total_volume + gender * app_diagnosis + gender * (amyg_l.sd + amyg_r.sd) + app_diagnosis * (amyg_l.sd + amyg_r.sd),df.sub.1)
LRT_amygsexdiag <- anova(lmamygsexdiag,lmamygsexdiag_null)

#Nuccleus Accumbens * Sex * Diagnosis
lmnucaccumbenssexdiag <- lm(cshq_sum.sd ~ scan_age + mori_total_volume + gender * app_diagnosis * ( nucaccumbens_l.sd + nucaccumbens_r.sd),df.sub.1)
lmnucaccumbenssexdiag_null <- lm(cshq_sum.sd ~ scan_age + mori_total_volume + gender * app_diagnosis + gender * (nucaccumbens_l.sd + nucaccumbens_r.sd) + app_diagnosis * (nucaccumbens_l.sd + nucaccumbens_r.sd),df.sub.1)
LRT_nucaccumbenssexdiag <- anova(lmnucaccumbenssexdiag,lmnucaccumbenssexdiag_null)

#Globus Pallidus * Sex * Diagnosis
lmgpsexdiag <- lm(cshq_sum.sd ~ scan_age + mori_total_volume + gender * app_diagnosis * ( gp_l.sd + gp_r.sd),df.sub.1)
lmgpsexdiag_null <- lm(cshq_sum.sd ~ scan_age + mori_total_volume + gender * app_diagnosis + gender * (gp_l.sd + gp_r.sd) + app_diagnosis * (gp_l.sd + gp_r.sd),df.sub.1)
LRT_gpsexdiag <- anova(lmgpsexdiag,lmgpsexdiag_null)

#Left Putamen * Sex * Diagnosis
lmputsexdiag <- lm(cshq_sum.sd ~ scan_age + mori_total_volume + gender * app_diagnosis * ( put_l.sd + put_r.sd),df.sub.1)
lmputsexdiag_null <- lm(cshq_sum.sd ~ scan_age + mori_total_volume + gender * app_diagnosis + gender * (put_l.sd + put_r.sd) + app_diagnosis * (put_l.sd + put_r.sd),df.sub.1)
LRT_putsexdiag <- anova(lmputsexdiag,lmputsexdiag_null)

#Pons * Sex * Diagnosis
lmponssexdiag <- lm(cshq_sum.sd ~ scan_age + mori_total_volume + gender * app_diagnosis * ( pons_l.sd + pons_r.sd),df.sub.1)
lmponssexdiag_null <- lm(cshq_sum.sd ~ scan_age + mori_total_volume + gender * app_diagnosis + gender * (pons_l.sd + pons_r.sd) + app_diagnosis * (pons_l.sd + pons_r.sd),df.sub.1)
LRT_ponssexdiag <- anova(lmponssexdiag,lmponssexdiag_null)

#Caudate * Sex * Diagnosis
lmcaudsexdiag <- lm(cshq_sum.sd ~ scan_age + mori_total_volume + gender * app_diagnosis * ( caud_l.sd + caud_r.sd),df.sub.1)
lmcaudsexdiag_null <- lm(cshq_sum.sd ~ scan_age + mori_total_volume + gender * app_diagnosis + gender * (caud_l.sd + caud_r.sd) + app_diagnosis * (caud_l.sd + caud_r.sd),df.sub.1)
LRT_caudsexdiag <- anova(lmcaudsexdiag,lmcaudsexdiag_null)

P_LRT_ROIbySEXbyDIAGNOSIS <- data.frame("LRT_hyposexdiag"=LRT_hyposexdiag[2,6],"LRT_hipposexdiag"=LRT_hipposexdiag[2,6],"LRT_thalamussexdiag"=LRT_thalamussexdiag[2,6],"LRT_amygsexdiag"=LRT_amygsexdiag[2,6],"LRT_nucaccumbenssexdiag"=LRT_nucaccumbenssexdiag[2,6],"LRT_gpsexdiag"=LRT_gpsexdiag[2,6],"LRT_putsexdiag"=LRT_putsexdiag[2,6],"LRT_ponssexdiag"=LRT_ponssexdiag[2,6],"LRT_caudsexdiag"=LRT_caudsexdiag[2,6])


# LRT ROI by SEX

#Hypothalamus * Sex
lmhyposex <- lm(cshq_sum.sd ~ scan_age + mori_total_volume + gender * app_diagnosis + gender * (hypothalamus_l.sd + hypothalamus_r.sd) + app_diagnosis * (hypothalamus_l.sd + hypothalamus_r.sd),df.sub.1)
lmhyposex_null <- lm(cshq_sum.sd ~ scan_age + mori_total_volume + gender * app_diagnosis + gender + app_diagnosis * (hypothalamus_l.sd + hypothalamus_r.sd),df.sub.1)
LRT_hyposex <- anova(lmhyposex,lmhyposex_null)

#Hippocampus * Sex
lmhipposex <- lm(cshq_sum.sd ~scan_age + mori_total_volume + gender * app_diagnosis + gender * (hippo_l.sd + hippo_r.sd) + app_diagnosis * (hippo_l.sd + hippo_r.sd),df.sub.1)
lmhipposex_null <- lm(cshq_sum.sd ~ scan_age + mori_total_volume + gender * app_diagnosis + gender + app_diagnosis * (hippo_l.sd + hippo_r.sd),df.sub.1)
LRT_hipposex <- anova(lmhipposex,lmhipposex_null)

#Thalamus * Sex
lmthalamussex <- lm(cshq_sum.sd ~ scan_age + mori_total_volume + gender * app_diagnosis + gender * (thalamus_l.sd + thalamus_r.sd) + app_diagnosis * (thalamus_l.sd + thalamus_r.sd),df.sub.1)
lmthalamussex_null <- lm(cshq_sum.sd ~ scan_age + mori_total_volume + gender * app_diagnosis + gender + app_diagnosis * (thalamus_l.sd + thalamus_r.sd),df.sub.1)
LRT_thalamussex <- anova(lmthalamussex,lmthalamussex_null)

#Amygdala * Sex
lmamygsex <- lm(cshq_sum.sd ~ scan_age + mori_total_volume + gender * app_diagnosis + gender * (amyg_l.sd + amyg_r.sd) + app_diagnosis * (amyg_l.sd + amyg_r.sd),df.sub.1)
lmamygsex_null <- lm(cshq_sum.sd ~ scan_age + mori_total_volume + gender * app_diagnosis + gender + app_diagnosis * (amyg_l.sd + amyg_r.sd),df.sub.1)
LRT_amygsex <- anova(lmamygsex,lmamygsex_null)

#Nuccleus Accumbens * Sex
lmnucaccumbenssex <- lm(cshq_sum.sd ~ scan_age + mori_total_volume + gender * app_diagnosis + gender * (nucaccumbens_l.sd + nucaccumbens_r.sd) + app_diagnosis * (nucaccumbens_l.sd + nucaccumbens_r.sd),df.sub.1)
lmnucaccumbenssex_null <- lm(cshq_sum.sd ~ scan_age + mori_total_volume + gender * app_diagnosis + gender + app_diagnosis * (nucaccumbens_l.sd + nucaccumbens_r.sd),df.sub.1)
LRT_nucaccumbenssex <- anova(lmnucaccumbenssex,lmnucaccumbenssex_null)

#Globus Pallidus * Sex
lmgpsex <- lm(cshq_sum.sd ~ scan_age + mori_total_volume + gender * app_diagnosis + gender * (gp_l.sd + gp_r.sd) + app_diagnosis * (gp_l.sd + gp_r.sd),df.sub.1)
lmgpsex_null <- lm(cshq_sum.sd ~ scan_age + mori_total_volume + gender * app_diagnosis + gender + app_diagnosis * (gp_l.sd + gp_r.sd),df.sub.1)
LRT_gpsex <- anova(lmgpsex,lmgpsex_null)

#Putamen * Sex
lmputsex <- lm(cshq_sum.sd ~ scan_age + mori_total_volume + gender * app_diagnosis + gender * (put_l.sd + put_r.sd) + app_diagnosis * (put_l.sd + put_r.sd),df.sub.1)
lmputsex_null <- lm(cshq_sum.sd ~ scan_age + mori_total_volume + gender * app_diagnosis + gender + app_diagnosis * (put_l.sd + put_r.sd),df.sub.1)
LRT_putsex <- anova(lmputsex,lmputsex_null)

#Pons * Sex
lmponssex <- lm(cshq_sum.sd ~ scan_age + mori_total_volume + gender * app_diagnosis + gender * (pons_l.sd + pons_r.sd) + app_diagnosis * (pons_l.sd + pons_r.sd),df.sub.1)
lmponssex_null <- lm(cshq_sum.sd ~ scan_age + mori_total_volume + gender * app_diagnosis + gender + app_diagnosis * (pons_l.sd + pons_r.sd),df.sub.1)
LRT_ponssex <- anova(lmponssex,lmponssex_null)

#Caudate * Sex
lmcaudsex <- lm(cshq_sum.sd ~ scan_age + mori_total_volume + gender * app_diagnosis + gender * (caud_l.sd + caud_r.sd) + app_diagnosis * (caud_l.sd + caud_r.sd),df.sub.1)
lmcaudsex_null <- lm(cshq_sum.sd ~ scan_age + mori_total_volume + gender * app_diagnosis + gender + app_diagnosis * (caud_l.sd + caud_r.sd),df.sub.1)
LRT_caudsex <- anova(lmcaudsex,lmcaudsex_null)


P_LRT_ROIbySEX <- data.frame("LRT_hyposex"=LRT_hyposex[2,6],"LRT_hipposex"=LRT_hipposex[2,6],"LRT_thalamussex"=LRT_thalamussex[2,6],"LRT_amygsex"=LRT_amygsex[2,6],"LRT_nucaccumbenssex"=LRT_nucaccumbenssex[2,6],"LRT_gpsex"=LRT_gpsex[2,6],"LRT_putsex"=LRT_putsex[2,6],"LRT_ponssex"=LRT_ponssex[2,6],"LRT_caudsex"=LRT_caudsex[2,6])


#LRT Sex * Diagnosis

#Hypothalamus; Sex * Diagnosis
lmhypo_sexbydiag <- lm(cshq_sum.sd ~ scan_age + mori_total_volume + gender * app_diagnosis + app_diagnosis * (hypothalamus_l.sd + hypothalamus_r.sd),df.sub.1)
lmhypo_sexbydiag_null <- lm(cshq_sum.sd ~ scan_age + mori_total_volume + gender + app_diagnosis * (hypothalamus_l.sd + hypothalamus_r.sd),df.sub.1)
LRT_hypo_sexbydiag <- anova(lmhyposex,lmhyposex_null)

#Hippocampus; Sex * Diagnosis
lmhippo_sexbydiag <- lm(cshq_sum.sd ~ scan_age + mori_total_volume + gender * app_diagnosis + app_diagnosis * (hippo_l.sd + hippo_r.sd),df.sub.1)
lmhippo_sexbydiag_null <- lm(cshq_sum.sd ~ scan_age + mori_total_volume + gender + app_diagnosis * (hippo_l.sd + hippo_r.sd),df.sub.1)
LRT_hippo_sexbydiag <- anova(lmhipposex,lmhipposex_null)

#Thalamus; Sex * Diagnosis
lmthalamus_sexbydiag <- lm(cshq_sum.sd ~ scan_age + mori_total_volume + gender * app_diagnosis + app_diagnosis * (thalamus_l.sd + thalamus_r),df.sub.1)
lmthalamus_sexbydiag_null <- lm(cshq_sum.sd ~ scan_age + mori_total_volume + gender + app_diagnosis * (thalamus_l.sd + thalamus_r),df.sub.1)
LRT_thalamus_sexbydiag <- anova(lmthalamussex,lmthalamussex_null)

#Amygdala; Sex * Diagnosis
lmamyg_sexbydiag <- lm(cshq_sum.sd ~ scan_age + mori_total_volume + gender * app_diagnosis + app_diagnosis * (amyg_l.sd + amyg_r.sd),df.sub.1)
lmamyg_sexbydiag_null <- lm(cshq_sum.sd ~ scan_age + mori_total_volume + gender + app_diagnosis * (amyg_l.sd + amyg_r.sd),df.sub.1)
LRT_amyg_sexbydiag <- anova(lmamygsex,lmamygsex_null)

#Nuccleus Accumbens; Sex * Diagnosis
lmnucaccumbens_sexbydiag <- lm(cshq_sum.sd ~ scan_age + mori_total_volume + gender * app_diagnosis + app_diagnosis * (nucaccumbens_l.sd + nucaccumbens_r.sd),df.sub.1)
lmnucaccumbens_sexbydiag_null <- lm(cshq_sum.sd ~ scan_age + mori_total_volume + gender + app_diagnosis * (nucaccumbens_l.sd + nucaccumbens_r.sd),df.sub.1)
LRT_nucaccumbens_sexbydiag <- anova(lmnucaccumbenssex,lmnucaccumbenssex_null)

#Globus Pallidus; Sex * Diagnosis
lmgp_sexbydiag <- lm(cshq_sum.sd ~ scan_age + mori_total_volume + gender * app_diagnosis + app_diagnosis * (gp_l.sd + gp_r.sd),df.sub.1)
lmgp_sexbydiag_null <- lm(cshq_sum.sd ~ scan_age + mori_total_volume + gender + app_diagnosis * (gp_l.sd + gp_r.sd),df.sub.1)
LRT_gp_sexbydiag <- anova(lmgp_sexbydiag,lmgp_sexbydiag_null)

#Putamen; Sex * Diagnosis
lmput_sexbydiag <- lm(cshq_sum.sd ~ scan_age + mori_total_volume + gender * app_diagnosis + app_diagnosis * (put_l.sd + put_r.sd),df.sub.1)
lmput_sexbydiag_null <- lm(cshq_sum.sd ~ scan_age + mori_total_volume + gender + app_diagnosis * (put_l.sd + put_r.sd),df.sub.1)
LRT_put_sexbydiag <- anova(lmput_sexbydiag,lmput_sexbydiag_null)

#Pons; Sex * Diagnosis
lmpons_sexbydiag <- lm(cshq_sum.sd ~ scan_age + mori_total_volume + gender * app_diagnosis + app_diagnosis * (pons_l.sd + pons_r.sd),df.sub.1)
lmpons_sexbydiag_null <- lm(cshq_sum.sd ~ scan_age + mori_total_volume + gender + app_diagnosis * (pons_l.sd + pons_r.sd),df.sub.1)
LRT_pons_sexbydiag <- anova(lmpons_sexbydiag,lmpons_sexbydiag_null)

#Caudate; Sex * Diagnosis
lmcaud_sexbydiag <- lm(cshq_sum.sd ~ scan_age + mori_total_volume + gender * app_diagnosis + app_diagnosis * (caud_l.sd + caud_r.sd),df.sub.1)
lmcaud_sexbydiag_null <- lm(cshq_sum.sd ~ scan_age + mori_total_volume + gender + app_diagnosis * (caud_l.sd + caud_r.sd),df.sub.1)
LRT_caud_sexbydiag <- anova(lmcaud_sexbydiag,lmcaud_sexbydiag_null)

P_LRT_SEXbyDIAGNOSIS <- data.frame("LRT_hypo_sexbydiag"=LRT_hypo_sexbydiag[2,6],"LRT_hippo_sexbydiag"=LRT_hippo_sexbydiag[2,6],"LRT_thalamus_sexbydiag"=LRT_thalamus_sexbydiag[2,6],"LRT_amyg_sexbydiag"=LRT_amyg_sexbydiag[2,6],"LRT_nucaccumbens_sexbydiag"=LRT_nucaccumbens_sexbydiag[2,6],"LRT_gp_sexbydiag"=LRT_gp_sexbydiag[2,6],"LRT_put_sexbydiag"=LRT_put_sexbydiag[2,6],"LRT_pons_sexbydiag"=LRT_pons_sexbydiag[2,6],"LRT_caud_sexbydiag"=LRT_caud_sexbydiag[2,6])

### run analyses for cshq_total

# Run multiple linear regression models for each brain region
library(broom)
df.sub.1$cshq_total.sd <- scale(log(df.sub.1$cshq_total), center = TRUE, scale = TRUE)

lmhypothallog <- lm(cshq_total.sd ~ gender + scan_age + mori_total_volume + app_diagnosis * (hypothalamus_l.sd + hypothalamus_r.sd),df.sub.1)
hypothalanova <- Anova(lmhypothallog,type = "II")


lmhippolog <- lm(cshq_total.sd ~ gender + scan_age + mori_total_volume + app_diagnosis * (hippo_l.sd + hippo_r.sd),df.sub.1)
hippoanova <- Anova(lmhippolog,type = "II")

lmthalamuslog <- lm(cshq_total.sd ~ gender + scan_age + mori_total_volume + app_diagnosis * (thalamus_l.sd + thalamus_r.sd),df.sub.1)
thalamusanova <- Anova(lmthalamuslog,type = "II")

lmamyglog <- lm(cshq_total.sd ~ gender + scan_age + mori_total_volume + app_diagnosis * (amyg_l.sd + amyg_r.sd),df.sub.1)
amyganova <- Anova(lmamyglog,type = "II")

lmnucaccumbenslog <- lm(cshq_total.sd ~ gender + scan_age + mori_total_volume + app_diagnosis * (nucaccumbens_l.sd + nucaccumbens_r.sd),df.sub.1)
nucaccumbensanova <- Anova(lmnucaccumbenslog,type = "II")

lmponslog <- lm(cshq_total.sd ~ gender + scan_age + mori_total_volume + app_diagnosis * (pons_l.sd + pons_r.sd),df.sub.1)
ponsanova <- Anova(lmponslog,type = "II")

lmcaudlog <- lm(cshq_total.sd ~ gender + scan_age + mori_total_volume + app_diagnosis * (caud_l.sd + caud_r.sd),df.sub.1)
caudanova <- Anova(lmcaudlog,type = "II")

lmgplog <- lm(cshq_total.sd ~ gender + scan_age + mori_total_volume + app_diagnosis * (gp_l.sd + gp_r.sd),df.sub.1)
gpanova <- Anova(lmgplog,type = "II")

lmputlog <- lm(cshq_total.sd ~ gender + scan_age + mori_total_volume + app_diagnosis * (put_l.sd + put_r.sd),df.sub.1)
putanova <- Anova(lmputlog,type = "II")

# Create a list of regression models for different brain regions
# Each model corresponds to a brain region and is stored with a descriptive name
models <- list(
 hypothalamus = lmhypothallog, # Model for hypothalamus
 hippocampus = lmhippolog, # Model for hippocampus
 thalamus = lmthalamuslog, # Model for thalamus
 amygdala = lmamyglog, # Model for amygdala
 nucaccumbens = lmnucaccumbenslog, # Model for nucleus accumbens
 pons = lmponslog, # Model for pons
 caudate = lmcaudlog, # Model for caudate
 gp = lmgplog, # Model for globus pallidus
 putamen = lmputlog # Model for putamen
)

# Extract regression coefficients (betas) and standard errors for main effects
# This focuses on the main effects of the brain regions (assumed to be 6th and 7th terms in each model)
main_results <- lapply(names(models), function(region_name) {
 tidy_mod <- broom::tidy(models[[region_name]]) %>%
 select(term, estimate, std.error) # Keep only the term, estimate, and std.error columns
 tidy_mod[c(6, 7), ] # Extract rows corresponding to main effects of the brain region
})

# Combine the results into a single data frame
df <- as.data.frame(do.call(rbind, main_results))

# Add an ID column to the data frame to maintain the order of terms
df$id <- 1:nrow(df)

# Extract p-values for main effects from the ANOVA results
# Note: Ensure the ANOVA objects (e.g., hypothalanova, hippoanova) are available in your environment
P_CSHQ_total <- data.frame(
 "hypothalamus_l.sd" = hypothalanova$`Pr(>F)`[5],
 "hypothalamus_r.sd" = hypothalanova$`Pr(>F)`[6],
 "hippo_l.sd" = hippoanova$`Pr(>F)`[5],
 "hippo_r.sd" = hippoanova$`Pr(>F)`[6],
 "thalamus_l.sd" = thalamusanova$`Pr(>F)`[5],
 "thalamus_r.sd" = thalamusanova$`Pr(>F)`[6],
 "amyg_l.sd" = amyganova$`Pr(>F)`[5],
 "amyg_r.sd" = amyganova$`Pr(>F)`[6],
 "nucaccumbens_l.sd" = nucaccumbensanova$`Pr(>F)`[5],
 "nucaccumbens_r.sd" = nucaccumbensanova$`Pr(>F)`[6],
 "pons_l.sd" = ponsanova$`Pr(>F)`[5],
 "pons_r.sd" = ponsanova$`Pr(>F)`[6],
 "caud_l.sd" = caudanova$`Pr(>F)`[5],
 "caud_r.sd" = caudanova$`Pr(>F)`[6],
 "gp_l.sd" = gpanova$`Pr(>F)`[5],
 "gp_r.sd" = gpanova$`Pr(>F)`[6],
 "put_l.sd" = putanova$`Pr(>F)`[5],
 "put_r.sd" = putanova$`Pr(>F)`[6]
)


P_CSHQ_total <- P_CSHQ_total %>%
 # Convert wide format to long format
 pivot_longer(
 cols = everything(),
 names_to = "term",
 values_to = "p_value"
 )

table_S2_fx <- merge(df, P_CSHQ_total, by = "term")
table_S2_fx <- table_S2_fx[order(table_S2_fx$id), ]

# Clean and reorganize the data for presentation in a table
new_df <- data.frame(table_S2_fx[, -4], row.names = table_S2_fx[, 4]) %>%
 mutate(
 # Extract the brain region name by removing the hemisphere suffix (_l.sd or _r.sd)
 region = str_remove(term, "_[lr]\\.sd$"),
 # Define the hemisphere based on the suffix in the term
 hemisphere = ifelse(str_detect(term, "_l\\.sd$"), "left", "right")
 ) %>%
 select(region, hemisphere, estimate, std.error, p_value) %>% # Select relevant columns
 mutate_at(vars(estimate, std.error), funs(round(., 2))) %>% # Round estimates and standard errors to 2 decimal places
 mutate_at(vars(p_value), funs(round(., 3))) # Round p-values to 3 decimal places

# Create summary table for interaction terms

main_results_int <- lapply(names(models), function(region_name) {
 tidy_mod <- broom::tidy(models[[region_name]]) %>%
 select(term, estimate, std.error)
 tidy_mod[c(8,9),]
})

df_interactions <- as.data.frame(do.call(rbind, main_results_int))


df_interactions$term <- str_remove(df_interactions$term , "app_diagnosisTD:")

df_interactions$id <- 1:nrow(df_interactions)


P_CSHQ_SUM_INTERACTION <-data.frame("hypothalamus_l.sd"=hypothalanova$`Pr(>F)`[7],"hypothalamus_r.sd"=hypothalanova$`Pr(>F)`[8],"hippo_l.sd"=hippoanova$`Pr(>F)`[7],"hippo_r.sd"= hippoanova$`Pr(>F)`[8],"thalamus_l.sd"= thalamusanova$`Pr(>F)`[7],"thalamus_r.sd"= thalamusanova$`Pr(>F)`[8],"amyg_l.sd"= amyganova$`Pr(>F)`[7],"amyg_r.sd"= amyganova$`Pr(>F)`[8],"nucaccumbens_l.sd"= nucaccumbensanova$`Pr(>F)`[7],"nucaccumbens_r.sd"= nucaccumbensanova$`Pr(>F)`[8],"pons_l.sd"= ponsanova$`Pr(>F)`[7],"pons_r.sd"= ponsanova$`Pr(>F)`[8],"caud_l.sd"= caudanova$`Pr(>F)`[7],"caud_r.sd"= caudanova$`Pr(>F)`[8],"gp_l.sd"= gpanova$`Pr(>F)`[7],"gp_r.sd"= gpanova$`Pr(>F)`[8],"put_l.sd"= putanova$`Pr(>F)`[7],"put_r.sd"= putanova$`Pr(>F)`[8])


P_CSHQ_SUM_INTERACTION <- P_CSHQ_SUM_INTERACTION %>%
 # Convert wide format to long format
 pivot_longer(
 cols = everything(),
 names_to = "term",
 values_to = "p_value"
 )

table_s2_int_fx <- merge(df_interactions, P_CSHQ_SUM_INTERACTION, by = "term")
table_s2_int_fx <- table_s2_int_fx[order(table_s2_int_fx$id), ]

new_int_df <- table_s2_int_fx %>%
 # Create new columns
 mutate(
 # Remove .sd suffix and extract hemisphere
 hemisphere = ifelse(str_detect(term, "_l\\.sd"), "left", "right"),
 # Clean region names by removing hemisphere suffixes
 region = str_remove(term, "_[lr]\\.sd$")
 ) %>%
 # Reorder columns
 select(region, hemisphere, estimate, std.error, p_value) %>%
 mutate_at(vars(estimate, std.error), funs(round(., 2))) %>%
 mutate_at(vars(p_value), funs(round(., 3)))
